# The common factor of executive functions measures nothing but speed of information uptake

**DOI:** 10.1007/s00426-023-01924-7

**Published:** 2024-02-19

**Authors:** Christoph Löffler, Gidon T. Frischkorn, Dirk Hagemann, Kathrin Sadus, Anna-Lena Schubert

**Affiliations:** 1 Institute of Psychology, Heidelberg University, Heidelberg, Germany; 2 Department of Psychology, University of Mainz, Mainz, Germany; 3 Department of Psychology, University of Zurich, Zurich, Switzerland

## Abstract

There is an ongoing debate about the unity and diversity of executive functions and their relationship with other cognitive abilities such as processing speed, working memory capacity, and intelligence. Specifically, the initially proposed unity and diversity of executive functions is challenged by discussions about (1) the factorial structure of executive functions and (2) unfavorable psychometric properties of measures of executive functions. The present study addressed two methodological limitations of previous work that may explain conflicting results: The inconsistent use of (a) accuracy-based vs. reaction time-based indicators and (b) average performance vs. difference scores. In a sample of 148 participants who completed a battery of executive function tasks, we tried to replicate the three-factor model of the three commonly distinguished executive functions shifting, updating, and inhibition by adopting data-analytical choices of previous work. After addressing the identified methodological limitations using drift–diffusion modeling, we only found one common factor of executive functions that was fully accounted for by individual differences in the speed of information uptake. No variance specific to executive functions remained. Our results suggest that individual differences common to all executive function tasks measure nothing more than individual differences in the speed of information uptake. We therefore suggest refraining from using typical executive function tasks to study substantial research questions, as these tasks are not valid for measuring individual differences in executive functions.

**Supplementary Information:**

The online version contains supplementary material available at 10.1007/s00426-023-01924-7.

## The common factor of executive function tasks measures nothing else but speed of information uptake

The umbrella term “executive functions” summarizes many top-down regulated abilities known under several synonyms, such as executive control, cognitive control, attentional control, and executive attention (Rey-Mermet et al., 2019). The most popular model of executive functions proposed by Miyake et al. (2000) includes three of these abilities: Shifting describes one’s ability to shift attention between different tasks or different mental sets; updating describes one’s ability to monitor memory contents and store new contents to the memory; inhibition describes one’s ability to block irrelevant information or interferences from the attentional focus (Friedman & Miyake, 2017; Friedman et al., 2008; Miyake & Friedman, 2012; Miyake et al., 2000; Rey-Mermet et al., 2018). This three-factor model has become the predominant model for describing and separating executive functions. Although the selection of abilities classified as executive functions by Miyake et al. (2000) was not exhaustive, it was based on practical and neuroanatomical considerations, as each of the three executive functions is associated with specific areas of the neocortex.

Several theoretical accounts of processes underlying individual differences in cognitive abilities claim that differences in executive functions determine differences in higher order cognitive abilities (Kane et al., 2008; Kovacs & Conway, 2016). Moreover, there is a large body of empirical findings reporting correlations between higher order cognitive processes (intelligence and working memory capacity [WMC]) and executive functions (e.g., Friedman et al., 2006, 2008, 2011).

To measure executive functions, researchers usually contrast two experimental conditions, i.e., one *condition with lower processing demands* and one *condition with greater processing demands*. For example, in an Arrow Flanker task (Eriksen & Eriksen, 1974), participants see an arrow in the center of a screen pointing to the left or right side. Participants have to indicate in which direction this arrow points. The arrow is shown amid further flanking stimuli. In the condition with lower processing demands (neutral condition), the central target arrow is surrounded by dashes not containing any directional or spatial information, which therefore have lower distracting effects on participants’ performance. In the condition with greater processing demands, the flanking stimuli consist of arrows pointing in the opposite direction of the target arrow (incongruent condition). Participants’ task is to ignore the irrelevant information of the flanker stimuli, which is more distracting in the condition with greater processing demands than in the neutral condition. This makes the decision in the condition with greater processing demands more difficult and leads to slower responses and higher error rates compared to the condition with lower processing demands. This decrease in performance between the two conditions, which are identical except for added demands on inhibition, indicates the specific strain on executive function demands (inhibitory processes). This strain occurs when participants have to ignore irrelevant flankers. In addition, performance in both conditions is also affected by *task-specific processes* as well as *task-general processes*. Following the logic of selective additivity, the performance decrement from the condition with less to the conditions with greater processing demands (e.g., the difference between reaction times [RTs] or accuracy rates) can be used to measure inhibitory demands, and individual differences in the performance decrement reflect individual differences in inhibition. Typical updating and shifting tasks are created following the same logic of selective additivity, allowing to analyze individual differences in the performance decrement. Because researchers have the choice between using RTs or accuracy rates as performance measures, there is much heterogeneity how individual differences in executive functions are assessed (von Bastian et al., 2020), even within single studies (Friedman et al., 2006, 2008; Himi et al., 2019, 2021; Ito et al., 2015; Krumm et al., 2009; Miyake et al., 2000; Schnitzspahn et al., 2013; Vaughan & Giovanello, 2010; Wongupparaj et al., 2015).

### Empirical findings on the three-factor model of executive functions

The three executive functions introduced by Miyake et al. (2000) represent distinct but interrelated factors (Friedman et al., 2006, 2008; Himi et al., 2019, 2021; Ito et al., 2015; Miyake et al., 2000; Schnitzspahn et al., 2013; Vaughan & Giovanello, 2010). Substantial correlations between the three latent factors raised the question of a higher-order factor of executive functions, often labeled as common executive functions. Hence, Friedman et al., (2008, 2011) further developed the model of three distinct factors into a model with two distinct factors of shifting and updating and an additional common factor of executive functions (see also Himi et al., 2019). This common factor supposedly represents the “ability to maintain task goals and goal-related information” (Miyake & Friedman, 2012, p. 3), which is considered as a general ability required in all cognitive tasks.

Despite the seemingly robust findings on the three executive functions model, recent research questions this factor structure and casts doubt on the existence of meaningful individual differences in specific executive functions, in particular inhibition (Frischkorn et al., 2019; Hedge et al., 2018; Hull et al., 2008; Karr et al., 2018; Klauer et al., 2010; Krumm et al., 2009; Rey-Mermet et al., 2018, 2019; Rouder & Haaf, 2019; Stahl et al., 2014; von Bastian et al., 2020). A recently published review by Karr et al. (2018) reported that previous studies showed evidence for both unidimensional and multidimensional factor structures of executive functions in adults. Karr et al. (2018) reanalyzed data from nine adult samples with different types of model composition to evaluate which type of model best describes executive functions data. They compared unidimensional models, nested-factor models (a special kind of bi-factor models), two-factor models, and three-factor models. Karr et al. (2018) found that none of the different model compositions was clearly superior and could be selected as the best model describing executive functions, although the authors observed slightly more evidence for nested-factor models than for the other model types. They attributed these inconsistencies in the dimensionality of models to a publication bias for well-fitting but possibly non-replicating models with underpowered sample sizes (Karr et al., 2018). This review clearly demonstrated that the factorial structure of executive functions is still an open research question.

Previous research did not only focus on the factor structure across, but also within specific executive functions. In particular, there is a lot of research on the factor structure of inhibition, with many papers demonstrating that inhibition tasks do not form a coherent latent factor (e.g., Krumm et al., 2009; Rey-Mermet et al., 2018, 2019; Rouder & Haaf, 2019; Stahl et al., 2014). For example, Rey-Mermet et al. (2018) used a battery of 11 inhibition tasks to analyze correlations between RT-based performance decrements, but could not find a coherent pattern of correlations between the performances in the different inhibition tasks. Instead, they found that inhibition abilities formed two correlated factors, one that reflected inhibition of prepotent responses and another that reflected inhibition of distractor interferences. Also, in a follow-up study using accuracy-based scores to measure inhibition, Rey-Mermet et al. (2019) could not observe a coherent factor structure among inhibition tasks. Likewise, Krumm et al. (2009) tried to replicate the three-factor model of executive functions using tasks from Miyake et al. (2000) with RT- and accuracy-based dependent variables, but they did not find a latent factor of inhibition. These results are exemplary for further studies that failed to find a coherent factor of inhibition even after accounting for trial-to-trial measurement noise (Rouder & Haaf, 2019; Stahl et al., 2014).

Further research suggested that the shared variance of executive function tasks is mainly driven by task-general process demands and not by demands specific to executive functions. For example, Frischkorn et al. (2019) separated the variance of experimental manipulations in executive function tasks from the shared variance of task-general processes, which are required in nearly every task and not specific to the experimental manipulation. The authors used adapted versions of a shifting task (Sudevan & Taylor, 1987), of an N-Back task (Scharinger et al., 2015), and of an Attentional Network task (Fan et al., 2002), and found that manipulation-specific variance (reflecting added executive demands) barely contributed to performance in executive function tasks. Instead, task-general processing abilities captured the majority of variance in task performance. Hence, performance in executive function tasks reflected task-general cognitive processes instead of specific executive functions (Frischkorn et al., 2019). In sum, executive function tasks, especially inhibition tasks, hardly measure a coherent construct or individual differences specific to executive functions. Instead, individual differences in general processing abilities explain most of the variance in performance in executive function tasks.

These inconsistent findings pose a problem for individual difference research and theoretical frameworks of executive functions: If it is impossible to find coherent factors of executive functions, it is impossible to assess covariations between these factors and other psychological constructs. A current literature review on attentional control and executive functions suggested that these inconstancies regarding the factor structure of executive functions may result from the psychometric properties of performance measures generated from executive function tasks (see von Bastian et al., 2020).

### The inconsistent use of dependent variables

There is much heterogeneity in how performance is assessed in executive function tasks (von Bastian et al., 2020). Usually, researchers use RT-based scores as measures in inhibition and shifting tasks, whereas they commonly use accuracy-based scores as measures in updating tasks (e.g., Friedman et al., 2006, 2008; Himi et al., 2019, 2021; Krumm et al., 2009; Miyake et al., 2000; Wongupparaj et al., 2015). We refer to such studies using different types of performance scores within their study designs as studies with *heterogeneous measurement scores.* In a recent review of 76 studies, von Bastian et al. (2020) showed that RT-based and accuracy-based scores were used more or less interchangeably to measure inhibition and shifting, whereas updating was typically assessed using accuracy-based scores. This inconsistent use of different types of performance scores can generate unexpected side effects because accuracy- and RT-based measures are often only weakly correlated, even in the same task (Hedge et al., 2018).

Furthermore, several studies measured individual differences in specific executive functions as difference scores, as the performance in the condition with higher task demands (e.g., RTs of the the incongruent condition in the Stroop task, Wongupparaj et al., 2015), or as the average performance over all conditions (e.g., averaged proportion correct across trials with different updating demands as updating scores; Miyake et al., 2000; for an overview, see also von Bastian et al., 2020). The issue with using either of the latter two measures is that other processes contribute to individual differences in performance in addition to the specific executive function demands. In particular, task-specific and task-general process parameters such as perceptual processing speed, the speed of decision-making, the speed of response preparation, and the speed of response execution contribute to individual differences in both condition-specific and task-general average performances. Consequently, using condition-specific or task-general average scores lowers the validity of the resulting measures if those are intended to only reflect specific executive functions. In consequence, correlations between these variables and other constructs do not necessarily reflect correlations between specific executive processes and other constructs but also of these other constructs with general performance parameters reflected in the measurement scores.

### Difference scores: high validity or further psychometric concerns?

Despite the seemingly greater face validity of difference scores in comparison to condition-specific or task-average scores, voices have been cautioning against the blind use of difference scores for two reasons. First, the use of difference scores relies on the assumption that each individual cognitive process added to an experimental task is independent of other processes and that each process has an additive effect on the performance measure (i.e., RTs and accuracy rates). In the Arrow Flanker task, for example, subtracting the RTs of the condition with lower processing demands (neutral condition) from the condition with greater processing demands (incongruent condition) should isolate specific inhibitory demands from more general processing demands affecting performance in both conditions (Donders, 1869). However, the assumption of additive processes has been challenged. For example, Miller and Ulrich (2013) introduced a model demonstrating that different processes contributing to RTs do not act independently from each other, but interact with each other, which is contrary to the assumption of their additivity. In a certain task, the specific executive function processes may, for example, interact with general processing demands. Following their reasoning, the subtraction of RTs from two conditions does not purely isolate executive function processes, because the influence of the interaction between general processing demands and executive function demands also remains in the difference score (Miller & Ulrich, 2013).

Second, some researchers caution against using difference scores because they tend to show low reliabilities (Ackerman & Hambrick, 2020; Draheim et al., 2019, 2023; Hedge et al., 2018; Miller & Ulrich, 2013; von Bastian et al., 2020; Weigard et al., 2021). Von Bastian et al. (2020) summarized the reliabilities of 406 measures of executive functions and found that the difference scores of inhibition tasks showed particularly low reliabilities with a mean reliability of 0.63 and a range from close to zero to close to one, whereas the reliability for shifting difference scores and updating scores were markedly higher (with mean reliabilities of 0.78). Low reliabilities are problematic for individual differences research, because they limit the strength of correlations with other measures (Cronbach & Furby, 1970; Spearman, 1904). Taken together, these issues of validity and reliability suggest that difference scores may not yield psychometrically sound measures of executive functions.

### Overcoming these problems

We summarized two problems of previous research measuring individual differences in executive functions, namely: (1) The inconsistent use of accuracy- and RT-based scoring methods, and (2) the psychometric problems of difference scores. Here we propose another analytical strategy to overcome these problems by combining cognitive modeling approaches with structural equation modeling. To address the first issue, we will use the drift rate parameter (*v*) of the diffusion model (Ratcliff, 1978), which is a mathematical model parameter that represents the speed of the evidence accumulation. For parameter estimation, the drift–diffusion model takes the distributions of correct as well as incorrect response times into account and thus integrates information about accuracies and RTs. To address the second issue, we will not control for general processing efficiency by controlling for performance in the condition with lower processing demands (by, e.g., calculating difference scores). Instead, we will control for general processing efficiency by using *elementary cognitive tasks*. The battery of three elementary cognitive tasks used in this study consists of three tasks with minimal executive demands often used in individual differences research (Frischkorn et al., 2019; Neubauer & Knorr, 1998; Schubert et al., 2015, 2017). We aim to use these tasks to measure individual differences in basic abilities of information processing largely free of executive demands, allowing us to control individual differences in performance in executive function tasks for individual differences in basic processing abilities (Frischkorn et al., 2019; Neubauer & Knorr, 1998; Schubert et al., 2015). This way, we overcome reliability problems of performance measures stemming from contrasting two conditions of the same task. These analytical choices will increase the likelihood of obtaining reliable and valid individual differences in executive functions.

#### Cognitive modeling to generate integrated measures of accuracies and RTs

**Fig. 1:**
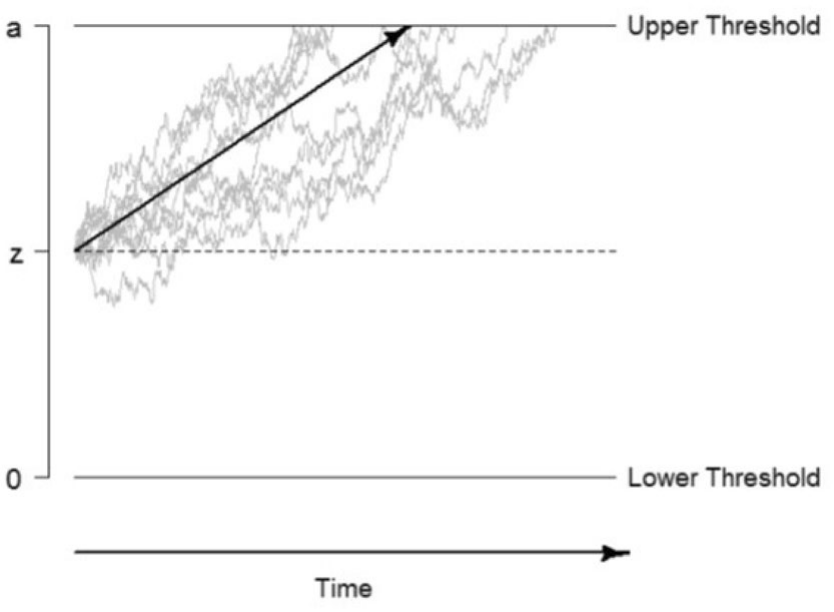
Graphical illustration of the drift–diffusion mode. *Note. *The decision process begins at the starting point *z*. Over the time more and more information will be accumulated until one of both thresholds is reached. The drift parameter *v* represents the strength and direction of the evidence accumulation process (represented by the black arrow). The parameter *a* describes the distance between both thresholds. The figure does not display the non-decision time *t*_*0*_*.* Figure with permission from Frischkorn and Schubert (2018), licensed under CC BY

To address the first problem—the inconsistent use of accuracies vs. RTs as indicator variables —, we used the drift parameter (*v*) of the drift–diffusion model to quantify participants’ task performances. The drift–diffusion model (Ratcliff, 1978) describes individuals’ cognitive processes in binary decision-making tasks, and distinguishes between decisional and non-decisional processes. By taking the whole intra-individual RT-distribution of correct and incorrect responses into account, we can estimate different parameters (for an illustration of the drift–diffusion model see Fig. 1). This means that the drift–diffusion model accounts for participants’ RTs and accuracy equally.

The model describes the decision-making process over time as a random walk during which information is taken up and evidence for a decision gets accumulated. In the drift–diffusion model, *v* describes the speed of information uptake and the strength and direction of the evidence accumulation process, that is, the average increase of evidence supporting one of the two choices per time unit. The decision process starts at the starting point (*z*), which can be used to model biases in decision making. During information uptake, the decision process approaches one of two decision thresholds. One threshold describes the correct and the other the alternative response. The boundary separation parameter (*a*) represents the distance between the two thresholds. In the course of time, *v* reaches one of two thresholds. Once it crosses a threshold, the decision process is terminated, and the response gets executed. The non-decision time parameter (*t*_*0*_) describes the speed of all non-decisional processes, such as the speed of motor-response execution and the speed of perceptional processes (see also Ratcliff et al., 2008; Voss et al., 2013).

Generally, participants’ drift rates in the standard drift–diffusion model are considered as measures of the speed of information uptake over time (Schmiedek et al., 2007; Voss et al., 2013) and are thus ability parameters that differ between individuals. Previous research on the psychometric properties of the drift rate parameter has shown that drift rates reflect both task-general and condition-specific processes (Lerche et al., 2020; Schubert et al., 2016). These specific components can be considered as specific abilities independent of the task-general speed of information uptake. In a previous study, Lerche et al. (2020) used a battery of different tasks measuring several domains of abilities (numerical, verbal, and figural) and separated the domain-specific processes (numerical, verbal, and figural abilities) from the domain-general processes (i.e., speed of information uptake) reflected in drift rates using bi-factor models. Following this logic, we aim to isolate variance specific to executive functions in drift rates after controlling for task-general processes (i.e., the speed of information uptake) on a latent level.

In contrast to the standard drift–diffusion model, recent research developed specific mathematical models to describe and measure the processes specific to executive functions more accurately: the shrinking spotlight diffusion model (White et al., 2011), the dual-stage two-phase model (Hübner et al., 2010), and the diffusion model for conflict task (Ulrich et al., 2015). However, in contrast to the standard drift–diffusion model, these newly developed models are specific to certain tasks (e.g., Arrow Flanker task or inhibition tasks) and not generalizable for all executive function tasks (e.g., updating and shifting tasks). In this study, our goal was to use one homogenous measurement score for all executive function tasks, which is why we chose the standard drift–diffusion model, fully aware that *v* represents only an approximation of the underlying processes.

First results of studies using drift rates to measure individual differences in executive functions are promising. For example, a recently published study showed that drift rates estimated from performances in seven different cognitive control tasks formed a common task-general factor of cognitive efficiency, which was related to self-reported cognitive control^1^ (Weigard et al., 2021). However, it remains unclear to what degree this factor reflected variance specific to executive processes and to what degree it reflected participants’ general speed of information uptake. Therefore, to capture individual differences in executive functions by drift rates, it is necessary to control for participants’ task-general speed of information uptake.

#### Structural equation modeling approach to avoid difference scores

To address the second problem of executive function research—the use of potentially problematical manifest difference scores—we proposed a structural equation modeling approach. In detail, we used the drift rates from the condition with greater processing demands of the different executive function tasks as *homogenous measurement scores* and controlled on the latent level for the influence of task-general processes, because the drift rates of the conditions with greater processing demands reflected not only processes specific to executive functions. We chose this structural equation modeling account because this method allows separating different kinds of variances and in particular distinguishing the variance in drift rates unique to executive function demands from the variance reflecting task-general processing demands. In our study, we therefore controlled the latent executive function factors for the influence of task-general speed of information uptake.

### The present study

The aim of the present study was to examine the factor structure of executive functions, whereby we attempted to address the two identified problems of executive function measures: (1) The inconsistent use of accuracy- and RT-based scoring methods and (2) the use of psychometrically unsatisfying difference scores. By applying a cognitive mathematical modeling approach and using *v* from the drift–diffusion model, we used a homogeneous scoring method for all executive function tasks. Additionally, we used structural equation models to separate the variance of task-general process demands from the variance of specific executive function demands.

Using data from 148 participants who completed a battery of different cognitive and experimental tasks, we first tried to replicate the factor structure of the seminal paper by Miyake et al. (2000) using accuracy rates and RT-based scores as performance measures (heterogenous measurement scores). Second, we estimated individuals’ task performances of all tasks with the drift rate parameter from the drift–diffusion model to integrate both RTs and accuracy rates simultaneously into one performance score. Third, we examined the factor structure of inhibition, updating, and shifting based on these parameter estimates. Fourth, we tested whether executive functions showed divergent validity to task-general speed of information uptake. Fifth, we evaluated the predictive validity of executive functions by relating them to individual differences in WMC and cognitive abilities. Taken together, our goal was to assess the factor structure of executive functions using error-free and valid measures of individual differences in inhibition, updating, and shifting.

## Materials and methods

### Openness and transparency

We provide access to the preprocessed data and the statistical analysis code used for this paper via the Open Science Framework (https://osf.io/6c4pu/). In addition, we provide access to the raw data and to the materials via the Open Science Framework (https://osf.io/4pvz3/; except for the materials of the BIS, which are commercially licensed).

### Statements and declarations

We declare no conflicts of interest. The study was approved by the ethics committee of the faculty of behavioral and cultural studies of Heidelberg University (reference number: Löf 2019/1–3). At the beginning of the first study session, participants signed an informed consent. All procedures were conducted in accordance with the Declaration of Helsinki (World Medical Association, 2013). This study was not preregistered.

### Participants

We recruited 151 participants from the general population via advertisements in different local newspapers, distribution of flyers, and acquisition by the participant pool of the department. Three participants declared their withdrawal from participation, which leads to a total sample of *N* = 148 participants (♀ 96, ♂ 51, one person declared no affiliation to either gender). We included participants between 18 and 60 years (*M*_age_ = 31.52, *SD*_age_ = 13.91) to generate a sample with heterogeneous cognitive abilities. Four participants stated having a different native language, but they were fluent in German. Thirty-nine percent of the sample had a university degree.

A minimum sample size of *N* = 95 would be needed to the hypothesis of close fit (H0: ε ≤ 0.05, H1: ε ≥ 0.08) as suggested by Browne and Cudeck (1992) for the most extensive structural equation model in this paper, displayed in Fig. 5 B (*df* = 166, alpha error: α = 0.05, power [1- β] = 0.80). The actual sample size of 148 participants yielded a power > 96% to test the hypothesis of close fit.^2^ Participants received 75 € and personal feedback about their performances in intelligence and working memory tests as compensation for participation.

### Materials

Table S1 in the supplementary materials shows the stimuli presentation times of the following 12 RT tasks. All computer-based tasks were programmed in MATLAB (The MathWorks Inc., Natick, Massachusetts) with the open source software package Psychtoolbox version 3.0.13 (Kleiner et al., 2007). We presented all the stimuli in the RT tasks in the center of the screen on a black background. In each task, we instructed the participants to respond as quickly and as accurately as possible. Before the experimental part of each task, participants worked on practice trials with feedback.

#### Inhibition

##### Stroop task

In each trial, participants saw one of four color words presented in one of four colors. The meaning of the word could be the same as the color in which the word was presented (congruent condition, 50% of the trials) or not (incongruent/inhibition condition, 50% of the trials). By pressing one of four keys on the keyboard, participants had to state the color of the word while they had to ignore its meaning (Stroop, 1935). Colored stickers on certain keys of the keyboard indicated the key mapping. We randomized the trials, with none of the conditions occurring more than three times in a row and none of the colors or words occurring twice in a row. Participants worked on 20 practice trials and 192 experimental trials.

##### Arrow flanker task

In each trial, one target arrow appeared in the center of the screen, pointing to the left or to the right direction. This target stimulus appeared in the middle of four flanker stimuli, two on each of both horizontal sides. The distractors could either point in the same direction as the target (congruent condition) or in the opposite direction (incongruent/inhibition condition; Eriksen & Eriksen, 1974). Participants had to indicate the side to which the target stimulus pointed while ignoring the distractors by pressing one of two keys on the keyboard. We randomized the trials, with none of the conditions or target directions occurring more than three times in a row. Participants worked first on 20 practice trials followed by 200 experimental trials.

##### Negative priming task

In each trial, two horizontal lines appeared on both sides next to the center of the screen. Subsequently, an X and an O appeared simultaneously on two of these lines. Participants had to indicate the position where the O appeared by pressing one of four keys while ignoring the X. In 50% of the trials, the O appeared at the position where the X appeared one trial before. To respond to an O shown at such a negatively primed position, participants had to redirect their attention to the positions previously associated with the distractor and overcome the transient residual inhibition (Tipper & Cranston, 1985). We randomized the trials, with none of the conditions (negatively primed vs. not negatively primed) occurring more than three times in a row and none of the stimuli appearing more than three times in a row on the same position. Participants worked on 20 practice trials and 192 experimental trials.

#### Updating

##### Keep track task

We adopted this task from the study by Miyake et al. (2000). Participants completed two blocks with different updating steps. The stimulus material consisted of four categories (letters, numbers, colors, geometric figures) and six stimuli within each category. Before each trial started, participants received an instruction about which of the four categories they had to keep track of. Depending on the block, they had to keep track of one or on three target-categories (updating steps: one or three). After that, participants saw a sequence of seven stimuli. This sequence contained stimuli from each of the four categories. Subsequently, a probe stimulus from one of the target categories followed. Participants had to indicate whether the probe stimulus was the last presented stimulus of the target category/categories (50% of the trials, matching condition) or not by pressing one of two keys on the keyboard. In 50% of the trials the target category was updated (updating condition). Within each block, participants worked on 10 practice trials and 96 experimental trials. We randomized the trials, with none of the conditions (matching and updating) and none of the target categories occurred more than three times in a row.

##### Running span task

We adopted this task from the study by Broadway and Engle (2010). In each trial, the stimuli of the memory and the stimuli of the updating set appeared sequentially in the center of the screen. Afterwards, participants saw a probe stimulus and had to decide whether this probe was part of the last three or last five stimuli, depending on the set size of the block. Participants completed two blocks with different set sizes. In both blocks the updating steps ranged from zero to three. Within the first block, the memory set consisted of three memory-letters followed by zero to three updating-letters. Within the second block, the memory set consisted of five memory-letters followed by zero to three updating-letters. Participants responded by pressing one of two keys on the keyboard. Half of the trials had zero updating steps. The other half of the trials included all one to three updating steps with equal frequency. In each block, participants worked on 10 practice trials and 120 experimental trials. We randomized the trials, with none of the updating steps and none of the probe stimuli occurring more than three times in a row.

##### N-back task

We adopted this task from the verbal working memory conditions of the task by Gevins et al. (1996). Participants completed three blocks, which included a different number of updating steps. In the first block, participants completed a 0-back task. Before the first block started, a target letter appeared, followed by 96 trials. In these trials either the target or a different letter was presented in the center of the screen. Specific target and non-target letters varied between participants. Participants had to decide whether the presented letter was the target or not by pressing one of two keys on the keyboard. Before the experimental part of the first block started, participants had to work on 20 practice trials. Data of the 0-back condition were not included in our analyses. In the second block, participants completed a 1-back task. In each trial, participants saw one of four letters in the center of the screen and had to decide whether or not this letter was equal to the stimulus that had appeared one trial before by pressing one of two keys on the keyboard. In total, participants completed 96 trials. In the third block, participants completed a 2-back task. In each trial, participants saw one of four letters in the center of the screen and had to decide whether or not this letter was equal to the stimulus that had appeared two trials before by pressing one of two keys on the keyboard. Again, we used 96 trials. Before the experimental part of the second and third block started, participants worked on 30 practice trials. Within each block the probe stimulus matched with the target stimulus in 50% of the trials (the stimulus one or two trials before = match condition). We randomized the trials, with none of the stimuli and none of the matching conditions occurring more than three times in a row.

#### Shifting

In each of the three shifting tasks, the color of the fixation cross at the beginning of each trial was the same as the color of the following probe stimulus.

##### Switching task

In each trial, a number between one and nine (except five) appeared either in red or in green in the center of the screen. Depending on the color of the presented stimulus, participants had to perform different tasks (Sudevan & Taylor, 1987). They had either to decide whether the number was less or more than five (red) or the number was odd or even (green) by pressing one of two keys on the keyboard. Both tasks appeared with equal frequency. In 50% of the trials, the task was the same as one trial before (repeat condition); in the other 50% of the trials, the color was different to the last trial (shifting condition). We randomized the trials, with none of the tasks and none of the conditions occurring more than three times in a row and none of the numbers appearing twice in a row. Participants worked on 10 task-pure practice trials for each of the two tasks, followed by 20 practice trials with both tasks intermixed. After the practice block, participants worked on 384 experimental trials.

##### Number letter task

In each trial, one number between one and nine (except five) together with one letter out of a set of eight letters appeared either in red or in green in the center of the screen. The letter set consisted of the letters A, E, I, U, G, K, M, and R. Depending on the color of the presented stimuli, participants had to perform different tasks. They had either to decide whether the number was less or more than five (red) or the letter was a consonant or a vocal (green) by pressing one of two keys on the keyboard (Rogers & Monsell, 1995). Both tasks appeared with equal frequency. Additionally, in 50% of the trials the task was the same as one trial before (repeat condition); in the other 50% of the trials, the color was different to the last trial (shifting condition). We randomized the trials, with none of the tasks and none of the conditions occurring more than three times in a row and none of the numbers and letters appearing twice in a row. Participants worked on 10 task-pure practice trials for each of the two tasks, followed by 20 practice trials with both tasks included. After the practice block, participants worked on 256 experimental trials.

##### Global local task

We adopted this task from the study by Miyake et al. (2000). In each trial, one of four geometrical shapes (circle, triangle, square, cross) appeared either in red or in green in the center of the screen. This figure was composed of small geometric shapes from the same set of shapes, better known as Navon-figures (Navon, 1977). The larger figure (global) and the smaller figure (local) could never have the same geometrical shape. Depending on the color, participants had to perform different tasks. They had either to identify the shape of the large figure (red) or the shape of the small figures (green) by pressing one of four keys on the keyboard. Both tasks appeared with equal frequency. In 50% of the trials the condition was the same as one trial before (repeat condition) in the other 50% of the trials the color was different to the last trial (shifting condition). We randomized the trials, with none of the tasks and none of the conditions occurring more than three times in a row and none of the large figures appearing twice in a row. Participants worked on 10 task-pure practice trials for each of the two tasks, followed by 20 practice trials with both tasks intermixed. After the practice block, participants worked on 384 experimental trials.

#### Processing speed

##### Two choice reaction time task

In each trial, participants had to focus on a centrally presented fixation cross, which was amid two quadratic frames. A plus sign appeared either in the left or in the right frame (e.g., Chen et al., 2012). Participants had to indicate whether the plus appeared in the left or in the right frame by pressing one of two response keys on the keyboard. The plus appeared on both sides with equal frequency. We randomized the trials, with none of the stimulus presentation sides repeating more than three times in a row. Participants worked on 20 practice trials, followed by 100 experimental trials.

##### Sternberg task

In each trial, five numbers between zero and nine appeared sequentially in the center of the screen. Following this sequence, a probe stimulus appeared and participants had to decide whether this probe was part of the formerly presented set or not (Sternberg, 1969) by pressing one of two response keys on the keyboard. In 50% of the trials the probe stimulus was part of the set (match condition). All numbers occurred with equal frequency as probe stimulus. We randomized the trials, with none of the conditions (match vs. no match) occurring more than three times in a row and none of the probe stimuli occurring twice in a row. Participants worked on 20 practice trials, followed by 100 experimental trials.

##### Posner task

In each trial, two letters appeared in the center of the screen. The stimulus set included the letters A, B, F, H, Q, a, b, f, h, q. Participants had to decide whether the meaning of the two letters was identical or not (e.g., Aa or AA = identical, AB or Ab = not identical; Posner & Mitchell, 1967). In 50% of the trials the letters had identical names. We randomized the trials, with none of the conditions (identical vs. not identical) and none of the letters occurring more than three times in a row. Participants worked on 20 practice trials, followed by 120 experimental trials.

#### Working memory capacity (WMC)

We used the memory updating task, the operation span task, the sentence span task, and the spatial short-term memory task from the working memory test battery by Lewandowsky et al. (2010) to assess participants’ WMC. In addition, we used the location-letter binding task by Wilhelm et al. (2013). All participants except five completed this letter binding task. For each of the different set sizes in the working memory tasks, we calculated participants’ mean proportion of correctly solved items as the dependent variable. Due to a programming error, we could not use the data of the spatial short-term memory task in our analyses.

#### Fluid intelligence

We used the short version of the Berlin Intelligence Structure Test (BIS, Jäger et al., 1997) as an assessment for fluid intelligence, which is a particularly suitable instrument for measuring higher-order cognitive abilities in a relatively short amount of time (about 50–60 min). The short version consists of a heterogeneous test battery including 15 different tasks. Four operation-related (processing capacity [PC], processing speed [PS], memory [M], creativity [C]) and three content-related (verbal, numerical, figural) components of intelligence can be assessed with the short version of the BIS. For our analyses, we calculated participants’ operation-related component scores by aggregating the normalized *z*-scores of all subtests measuring the respective component. Participants had a mean IQ of 96 (*SD* = 15.86).

### Procedure

Participants completed three measurement occasions within one year. At the beginning of the first session, participants signed an informed consent and completed the Ishihara-Test (Ishihara, 2000) to rule out that they were colorblind. Following that, we prepared participants’ EEG and seated them in a dimly lit cabin during the first and second measurement occasions. The EEG data are not reported in the current paper (see Sadus et al., 2023; Schubert et al., 2022a, 2022b). Subsequently, participants worked on the 12 tasks in the following order. Measurement occasion one: Sternberg task, Arrow Flanker task, Global Local task, N-Back task, Switching task, and Stroop task. Measurement occasion two: Running Span task, Two Choice Reaction Time task, Number Letter task, Negative Priming task, Keep Track task, and Posner task. In addition, participants completed a questionnaire about their demographical data at the end of the first occasion. Each occasion lasted approximately 3.5 h. To avoid between-subjects error variance by balancing the task order, we decided to present all tasks for all participants in the same order, well knowing that this procedure might result in fatigue, reduced motivation, or sequence effects systematically affecting performance measures (Goodhew & Edwards, 2019). During the third measurement occasion, participants first completed the intelligence test followed by the working memory test battery and the letter binding task. In addition, participants also completed two short tests measuring their higher-order cognitive abilities, a mind-wandering questionnaire, and a pretzel task (these data are not reported here).

### Data analysis

We used the statistics software R—version 4.1.0 (R. Core Team, 2022) for data preprocessing and analyses and used the following packages: For preparation and data management the package “tidyverse” (Wickham et al., 2019), for descriptive statistics the package “psych” (Revelle, 2020), for correlations the package “Hmisc” (Harrell, 2019), for structural equation model analyses the package “lavaan” (Rosseel, 2012), for confidence interval estimations the package “MBESS” (Kelley, 2007), and for the preparation of the correlation matrices the package “patchwork” (Pedersen, 2020).

### Outlier analysis and data processing

Before we conducted the main analyses, we performed univariate intra- and inter-individual outlier analyses. The procedure was identical for each participant and variable. The detected outliers (trials or participants) were excluded only from the corresponding conditions of the respective task.

For the intra-individual outlier analysis, we applied the following steps to each condition in each executive function and processing speed task. Initially, responses faster than 150 ms were discarded. Subsequently, we logarithmized and *z*-transformed the RT variables for each participant and removed the trials with *z*-values greater than 3 or smaller than -3. On average, 0.69% of the trials were removed within each condition of the 12 reaction time tasks (range: 0.33% to 1.06%).

Next, we conducted inter-individual outlier analyses based on both RT and accuracy scores for each condition in the twelve tasks. Participants with accuracy scores below the guessing probability threshold were discarded. This threshold was determined based on the number of trials and response options of the corresponding condition, assuming a binomial distribution. In addition, we identified mean RTs or logit-transformed accuracy values that deviated from the average by more than 3 standard deviations as inter-individual outliers. These participants were removed from the corresponding task.

Following the outlier analyses, we modified the data for subsequent analyses according to our requirements. This involved estimating participants’ drift–diffusion model parameters for all conditions of all tasks separately (details below). In addition, to replicate the model of three interrelated executive functions by Miyake et al. (2000), we removed all incorrect trials and calculated participants’ RT-difference scores for the shifting and inhibition tasks, their mean RTs for the inhibition tasks, and their arcsine-transformed probability scores for the updating tasks. Before we inserted the variables in the structural equation models, we discarded the values deviating from the average by more than three standard deviations. Accumulated over all these steps of the inter-individual outlier detection, we removed, on average, 3.63% of the participants within each of the variables (range: 0.70% to 7.09%).

#### Drift–diffusion modeling

We fitted the diffusion model parameters with *fast-dm-30* (Voss et al., 2015) using the Kolmogorov–Smirnov criterion for optimization. For each participant, we estimated (*v*), the boundary separation (*a*), the non-decision time (*t*_*0*_), and the inter-trial-variability of the non-decision time (*st*_*0*_) in the conditions with greater processing demands of the executive function tasks. Further, we followed the recommendations of Lerche and Voss (2016) and fixed all other parameters to zero except the starting point* z*, which we centered between the two decision thresholds (*z* = 0.5).

Subsequently, we assessed if the drift–diffusion models provided a good account to the observed data by evaluating the models using simulated RT and accuracy data based on model parameters. The correlations between the observed and predicted scores were between *r* = 0.94 and *r* = 0.99 for the RTs in the 25th, 50th and 75th percentile of the RT distributions and between *r* = 0.47 and *r* = 0.87 for the overall accuracy scores (except for the accuracies in the Two Choice Reaction Time task, *r* = 0.05; see for further discussion the limitations section), which indicated that there was overall no evidence for a systematically biased model prediction. For a visual inspection of the model fits see Fig. S1 to Fig. S4 in the supplementary materials.

In three decision tasks, participants had to respond by pressing four instead of two keys, which is not a binary choice in the classical way (Stroop task, Negative Priming task, Global Local task). However, Voss et al. (2015) argued that diffusion modeling of tasks with more than two response keys is possible under some assumptions: The responses have to be re-coded as either correct or incorrect, drift rates should not differ between stimulus types, there should be no bias in response behavior, and these tasks should have a sufficient number of errors (Voss et al., 2015). The three tasks with more than two response options in our study met these assumptions. In addition, the parameter recovery indicated no systematically lower predictions of these scores compared with the classical binary choice tasks.

#### Structural equation modeling

First, we wanted to replicate the original model of three interrelated executive function factors by Miyake et al. (2000). For this, we used similar scores for the manifest variables as in the original study. Second, we estimated the three-factor model of executive functions, with drift parameters difference scores. Therefore, the drift rate parameters were estimated separately for the two conditions of each task, while the other parameters of the drift–diffusion model were kept constant. Afterwards, we contrasted the drift parameters to get the drift differences scores for each of the nine executive function tasks and inserted these difference scores as indicators in the three-factor model of executive functions. Third, we estimated the drift parameters only for the conditions with greater processing demands, which were used as indicators for the following analyses and models. Again, we specified the three-factor model of executive functions based on these drift rate parameters. Fourth, we estimated a model with a second-order factor as well as a model with a first-order factor of *common executive functions* and examined in the following step the relations of this common factor to higher-order cognitive abilities. Fifth, to control for task-general speed of information uptake, we regressed the common factor of executive functions on a task-general *speed* factor estimated from three elementary cognitive tasks and examined again the relations of the latent variables to intelligence and WMC.

To account for missing data, we used full information maximum likelihood (FIML). We fixed one of the loadings of each factor to one and estimated the variances of the latent factors. The goodness-of-fit was evaluated by the comparative fit index (CFI; Bentler, 1990) and the root mean square error of approximation (RMSEA; Browne & Cudeck, 1992). Following the recommendations by Browne and Cudeck, (1992) as well as Hu and Bentler (1999), we considered CFI values > 0.90 and RMSEA values ≤ 0.08 as an acceptable model fit and CFI values > 0.95 and RMSEA values ≤ 0.06 as good model fit. In direct model comparisons, AIC differences ≥ 10 indicated substantial advantages (Burnham & Anderson, 2002). We assessed the statistical significance of model parameters with the two-sided critical ratio test.

## Results

First, we tried to replicate the model of three distinct but interrelated factors of executive functions by Miyake et al. (2000) with heterogeneous measurement scores. Afterwards, we examined the factor structure of executive functions and its relation to higher-order cognitive abilities using the drift parameters of the drift–diffusion model as homogenous measurement scores. The descriptive statistics of the heterogeneous measurement scores are displayed in Table 1. We found large variations of reliability estimates for the heterogeneous measurement scores (see Table 1). The reliability estimates were excellent for the inhibition tasks if performance was measured by mean RTs and poor to acceptable if performance was measured by RT-differences scores. Reliabilities varied from moderate to good in the updating tasks, where performance was measured by arcsine-transformed proportion correct scores. Reliabilities were poor in the shifting tasks, where performance was measured by RT-difference scores. The correlations between the heterogeneous measurement scores are shown in Table S2 in the supplementary materials.

**Table 1:** Descriptive statistics of the heterogeneous measurement scores

Task name	Measurement score	Mean	*SD*	Reliability
Negative priming task	RT difference	0.02	0.02	0.28^a^
Flanker task	RT difference	0.03	0.02	0.66^a^
Stroop task	RT difference	0.11	0.06	0.77^a^
Negative priming task, priming cond	RT	0.61	0.11	0.99^a^
Flanker task, incong. cond	RT	0.50	0.08	0.99^a^
Stroop task, incong. cond	RT	0.82	0.14	0.98^a^
Keep track task, updating cond	a. t. proportion correct (percent correct)	1.29 (91.40)	0.11 (6.38)	0.57^a^
Running span task, updating cond	a. t. proportion correct (percent correct)	1.28 (90.99)	0.08 (4.85)	0.64^a^
N-back task	a. t. proportion correct (percent correct)	1.21 (86.29)	0.13 (8.64)	0.85^a^
Number letter task	RT difference	0.06	0.07	0.68^a^
Switching task	RT difference	0.05	0.06	0.45^a^
Global local task	RT difference	0.09	0.07	0.41^a^
Two choice RT task	RT	0.38	0.04	0.99^a^
Sternberg task	RT	0.91	0.22	0.98^a^
Posner task	RT	0.71	0.13	0.99^a^
Memory updating	Percent correct	63	20	0.88^b^
Binding	Percent correct	86	11	0.82^b^
Operation span	Percent correct	78	13	0.89^b^
Sentence span	Percent correct	84	11	0.87^b^
BIS-PC	Scales-scores	101.61	7.12	0.75^b^
BIS-PS	Scales-scores	101.14	7.15	0.49^b^
BIS-M	Scales-scores	98.59	7.16	0.58^b^
BIS-C	Scales-scores	98.15	6.97	0.45^b^

*Note.* Heterogeneous measurement scores; a. t. = arcsine-transformed; BIS-PC = processing capacity scale of the Berlin Intelligence Structure Test; BIS-PS = processing speed scale of the Berlin Intelligence Structure Test; BIS-M = memory scale of the Berlin Intelligence Structure Test; BIS-C = creativity scale of the Berlin Intelligence Structure Test

^a^Reliability estimates are based on Spearman–Brown corrected odd–even split correlations

^b^Reliability estimates are based on Cronbach’s α; RT-values are displayed in seconds; proportion correct = arcsine-transformed proportion correct scores

**Fig. 2:**
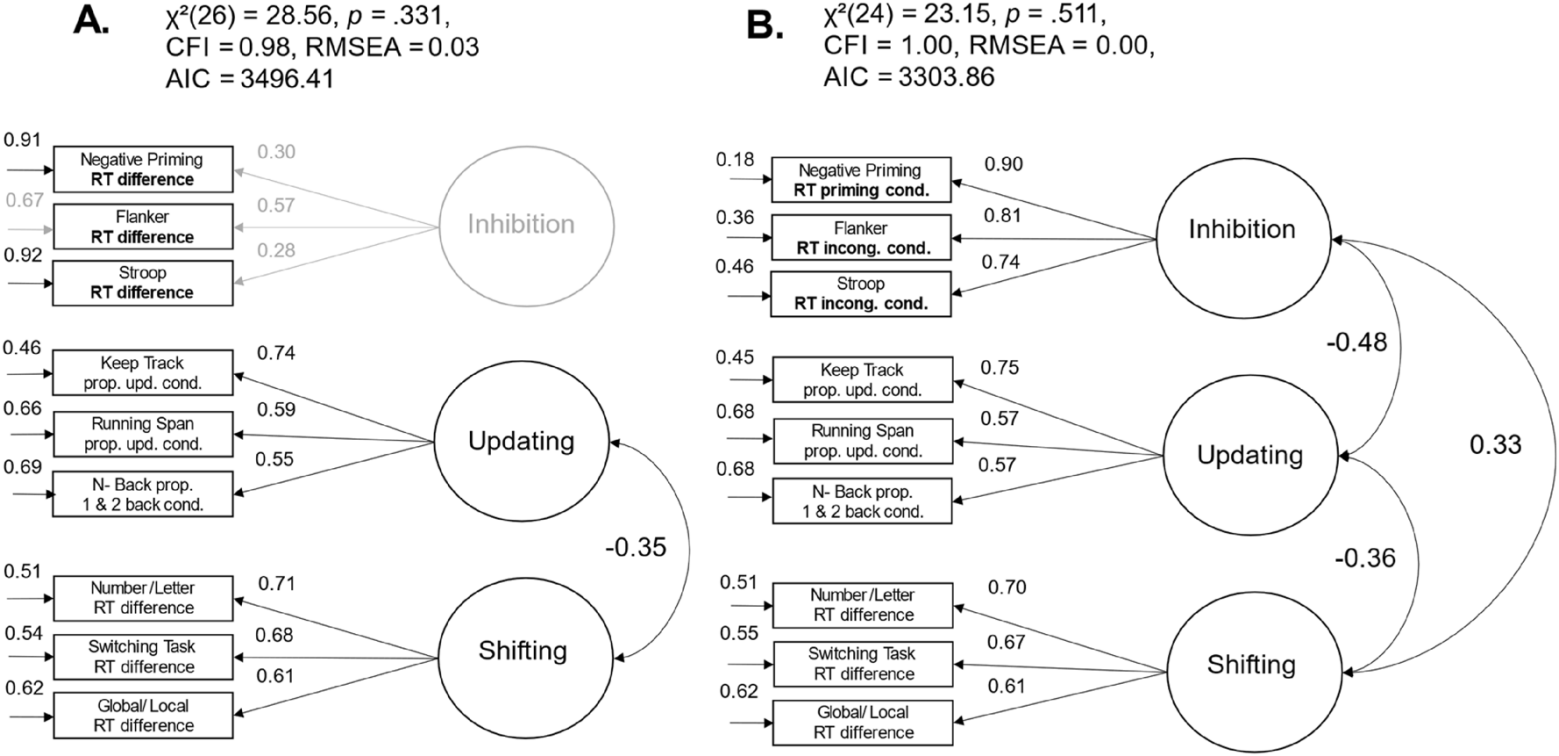
Three-factor models of executive functions with heterogeneous measurement scores. *Note*. The standardized path weights, the unstandardized residual variances, and the correlation coefficients are shown next to the paths; non-significant estimators are grayed out

We specified the model of three distinct but interrelated factors of executive functions to replicate the model by Miyake et al. (2000) and compared how the factor structure of drift rates differed from the factor structure of heterogeneous measurement scores. In the original model by Miyake et al. (2000), they used RT-based difference scores between incongruent and congruent conditions to measure participants’ abilities in inhibition, RT-based difference scores between shifting and repeat conditions to measure participants’ shifting abilities, and arcsine-transformed proportion correct scores to measure participants’ updating performance. When we used these measurement procedures in our own data, the model provided a good account of the data, *χ*^2^(26) = 28.56, *p* = 0.331, CFI = 0.98, RMSEA = 0.03, 95% CI [0.00, 0.08]; see Fig. 2A). However, we could not find significant variance in the latent inhibition factor with RT difference scores, σ^2^ = 0.09, *p* = 0.431, 95% CI [− 0.13, 0.30]. Therefore, we decided to use the mean RT-scores of the conditions with greater processing demands, the inhibition conditions, to examine individual differences in inhibition. The corresponding model provided an excellent account of the data, *χ*^2^(24) = 23.15, *p* = 0.511, CFI = 1.00, RMSEA = 0.00, 95% CI [0.00, 0.07]; see Fig. 2B). The three latent executive function factors were moderately correlated. Participants who were less distracted by irrelevant information (shorter RTs in inhibition tasks) showed better updating abilities, *r* = − 0.48, *p* < 0.001, 95% CI [− 0.66, − 0.30], and lower shifting costs, *r* = 0.33, *p* = 0.004, 95% CI [0.13, 0.53]. Moreover, participants with better updating abilities showed lower shifting costs, *r* = − 0.36; *p* = 0.010, 95% CI [− 0.59, − 0.13].

We were (mostly) able to replicate the original model of three distinct but interrelated factors of executive functions. In the next step, we wanted to examine the factor structure of executive functions by using drift rates instead of RT-/accuracy-based performance scores.

Conducting the analyses based on the drift rate parameters, we first used drift rate difference scores to examine the reliability and factor structure of executive functions. However, the covariance matrix of the latent variables was not positive definite and the model did not converge. Furthermore, the Spearman–Brown corrected odd–even correlations indicated insufficient reliabilities or even inadmissible estimates for the drift rate difference scores (ranging from -0.12 to 0.66). In consequence, we can conclude that even for drift rates, the difference scores tended to be unreliable and did not prove to be useful indicators measuring individual differences in executive functions. This highlights the limited utility of difference scores in executive function research and underscores our strategy to examine drift rates of the conditions with greater processing demands and to disentangle the sources of variance at the latent level. Table 2 shows the descriptive statistics for drift rates from the conditions with greater processing demands of the nine executive function tasks and of the three processing speed tasks and (see Table S3 in the supplementary materials for the descriptive statistics of the other estimated drift–diffusion model parameters).

**Table 2:** Descriptive statistics of drift rates

	Measurement score	Mean	SD	Reliability
Negative priming task, priming cond	Drift parameter *v*	3.62	0.94	0.47
Flanker task, incong. cond	Drift parameter *v*	5.05	1.32	0.57
Stroop task, incong. cond	Drift parameter *v*	2.60	0.74	0.48
Keep track, updating cond	Drift parameter *v*	1.69	0.62	0.81
Running span, updating cond	Drift parameter *v*	1.78	0.58	0.67
N-back task	Drift parameter *v*	1.74	0.46	0.83
Number letter task, shifting cond	Drift parameter *v*	2.29	0.92	0.89
Switching task, shifting cond	Drift parameter *v*	2.16	0.85	0.90
Global local task, shifting cond	Drift parameter *v*	1.65	0.51	0.79
Two choice RT task	Drift parameter *v*	6.25	1.58	0.70
Sternberg task	Drift parameter *v*	2.35	0.71	0.45
Posner task	Drift parameter *v*	3.27	0.74	0.53

*Note.* Drift rates *v* as measurement scores; reliability estimates are based on Spearman–Brown corrected odd–even split correlations

Overall, the reliabilities of drift rates were on average slightly smaller but comparable to RT- and accuracy-based performance measures. They showed a broad range from poor to good reliabilities. The small difference between the reliabilities of heterogeneous and homogeneous measurement scores are mainly driven by the RT average scores of the inhibition- and elementary cognitive tasks, which usually show very high reliabilities. In comparison to the reliabilities reported in Table 1, reliabilities were higher for updating and shifting tasks, but lower for inhibition tasks. The correlations between the drift rate parameters of each task are shown in Table S4 in the supplementary materials.

**Fig. 3:**
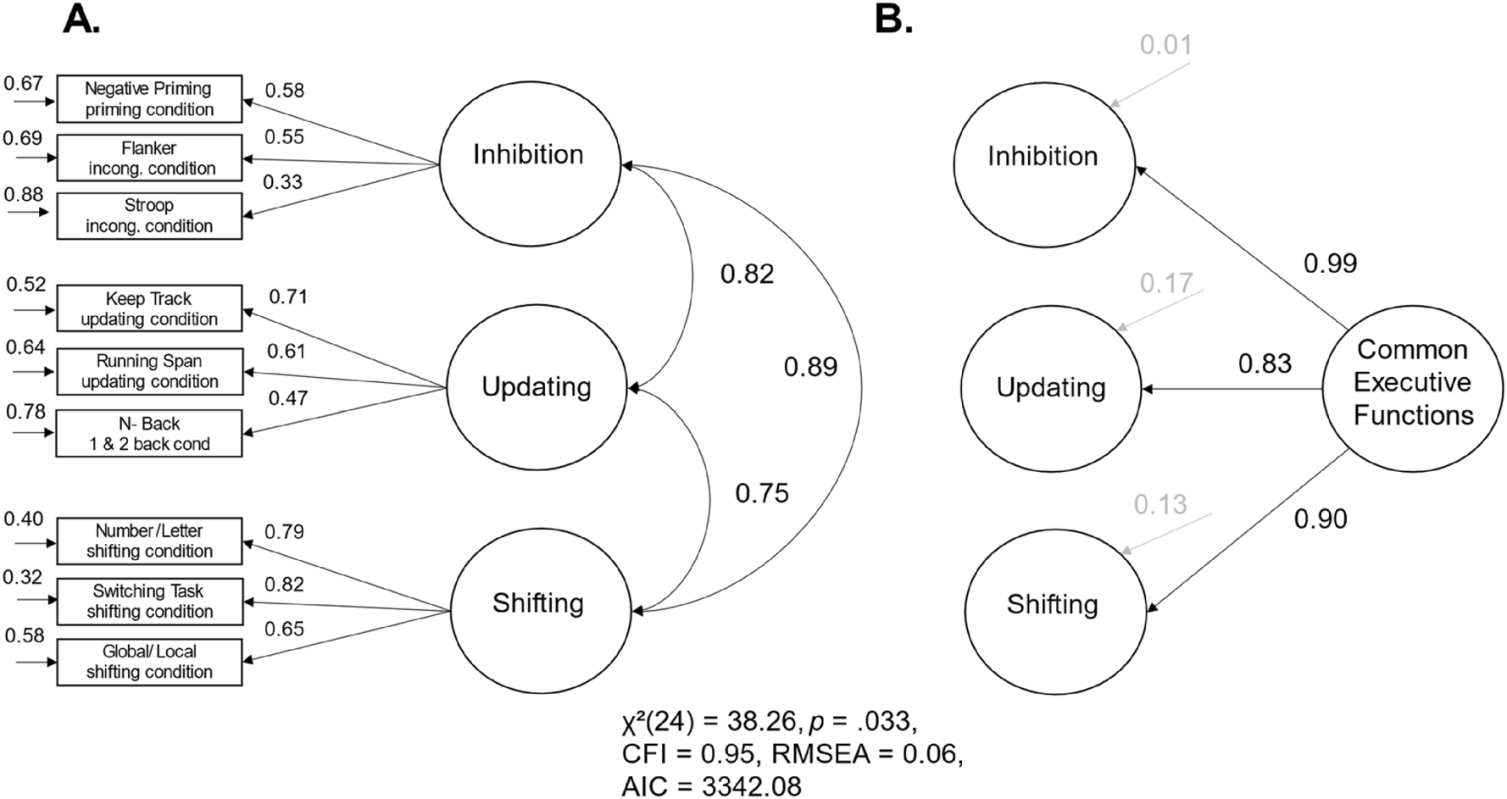
Three-factor models of executive functions with drift rates as homogenous measurement scores. *Note*. The standardized path weights, the unstandardized residual variances, and the correlation coefficients are shown next to the paths; non-significant estimators are grayed out

Subsequently, we specified the model of three distinct, but interrelated factors based on drift rates instead of the heterogeneous measurement scores (see Fig. 3A). The model provided a good fit of the data, *χ*^2^(24) = 38.26, *p* = 0.033, CFI = 0.95, RMSEA = 0.06, 95% CI [0.00, 0.11]. The three latent executive function factors were highly correlated. Participants with higher drift rates in inhibition tasks showed higher drift rates in updating tasks (*r* = 0.82, *p* < 0.001, 95% CI [0.59, 1.06]) as well as higher drift rates in shifting tasks, *r* = 0.89, *p* < 0.001, 95% CI [0.70, 0.1.08]. Furthermore, participants with higher drift rates in updating tasks showed higher drift rates in shifting tasks, *r* = 0.75, *p* < 0.001, 95% CI [0.60, 0.90]. Taken together, we were also able to find the three latent factors of executive functions by using drift rates instead of heterogenous measurement scores. The positive manifold in the correlations between the three latent factors suggests a hierarchical factor structure with a higher-order factor of executive functions or a one-factor solution with a common factor of executive functions on the first level.

In consequence, we introduced a higher-order factor of executive functions (*common executive functions*) in our model with drift rates as manifest variables (see Fig. 3 B). The model fit was equivalent to the model just described, in which the latent first-order factors were correlated. However, after introducing this second-order factor of executive functions, there was no remaining significant residual variance specific to each of the three executive functions (inhibition: residual variance σ^2^ = 0.01, *p* = 0.942, 95% CI [− 0.14, 0.15]; updating: residual variance σ^2^ = 0.17, *p* = 0.061, 95% CI [− 0.01, 0.34]; shifting: residual variance σ^2^ = 0.13, *p* = 0.133, 95% CI [− 0.04, 0.30]). If we fixed the residual variances to zero, the model fit deteriorated only slightly, but not above the critical AIC difference proposed by (Burnham & Anderson, 2002), ∆ AIC = 7.60, *χ*^2^(27) = 51.86, *p* = 0.003, CFI = 0.91, RMSEA = 0.08, 95% CI [0.04, 0.12]. Inhibition, updating, and shifting, as measured with *v*, were fully explained by the higher-order factor.

**Fig. 4:**
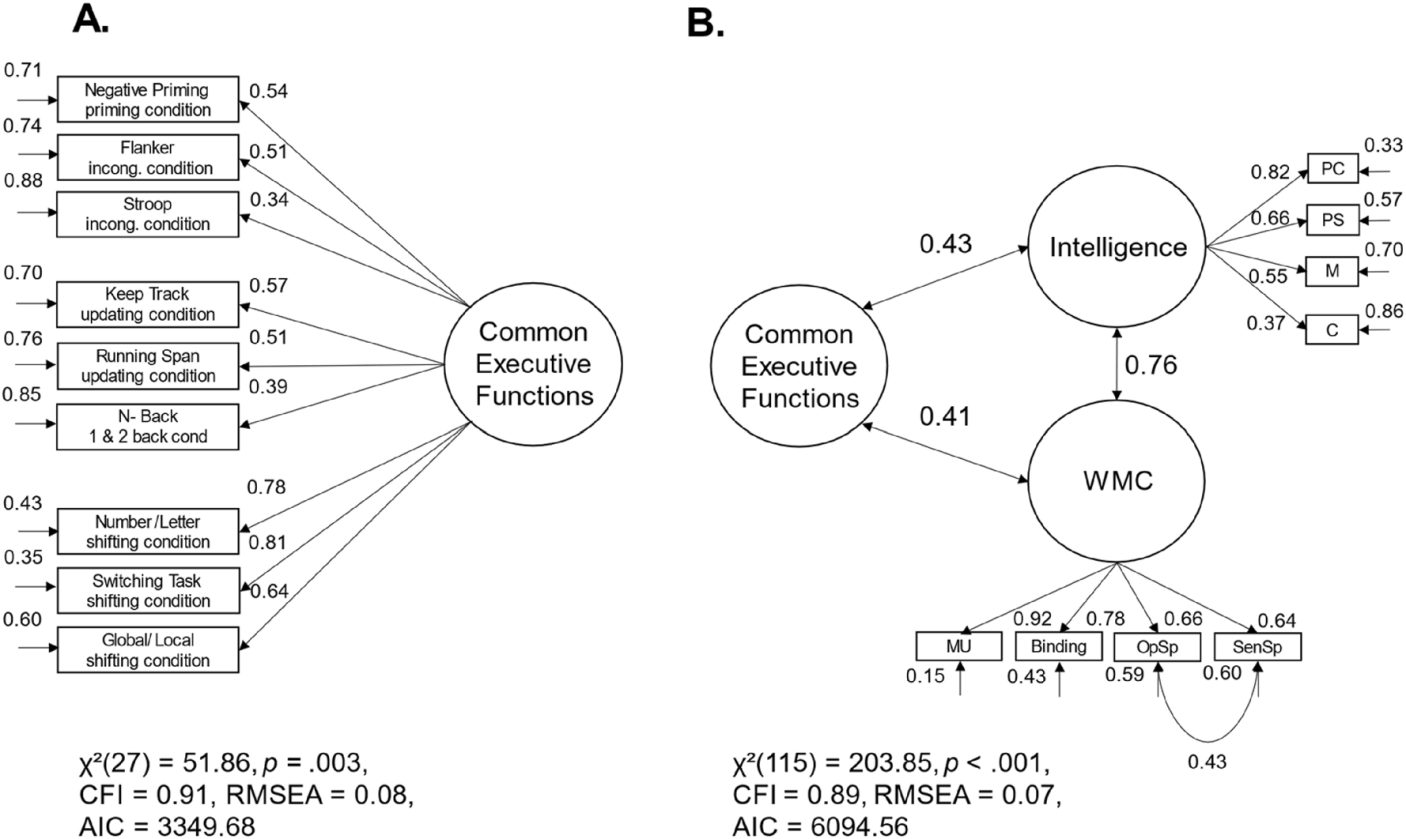
Models with one common factor of executive functions with drift rates as homogenous measurement scores. *Note*. The standardized path weights, the unstandardized residual variances, and the correlation coefficients are shown next to the paths; MU = memory updating; BIS-PC = processing capacity scale of the Berlin Intelligence Structure Test; BIS-PS = processing speed scale of the Berlin Intelligence Structure Test; BIS-M = memory scale of the Berlin Intelligence Structure Test; BIS-C = creativity scale of the Berlin Intelligence Structure Test; WMC = working memory capacity

The non-significant residual variances of the three latent factors of executive functions on the first level suggested a one-factor structure of executive functions. Therefore, we specified a model with only one latent common executive functions factor (see Fig. 4A). The model fit was equivalent to the model just described, in which the residual variances of the first-order factors were set to zero. Our findings suggest that the drift parameters of the different executive function tasks represented individual differences in one common executive ability.

In the next step, we examined the correlations between the common factor of executive functions and higher-order cognitive abilities. Please note that in this model, we have not yet controlled for task-general speed of information uptake. We introduced Intelligence and WMC as latent factors into the model (see Fig. 4B). The model provided an almost acceptable fit of the data, *χ*^2^(115) = 203.85, *p* < 0.001, CFI = 0.89, RMSEA = 0.07, 95% CI [0.05, 0.09]. Intelligence and WMC factors were highly correlated, *r* = 0.76, *p* < 0.001, 95% CI [0.64, 0.88]. Individual differences in executive functions were moderately related to intelligence (*r* = 0.43, *p* < 0.001, 95% CI [0.25, 0.62]) and WMC, *r* = 0.41, *p* = 0.001, 95% CI [0.25, 0.58].^3^

**Fig. 5:**
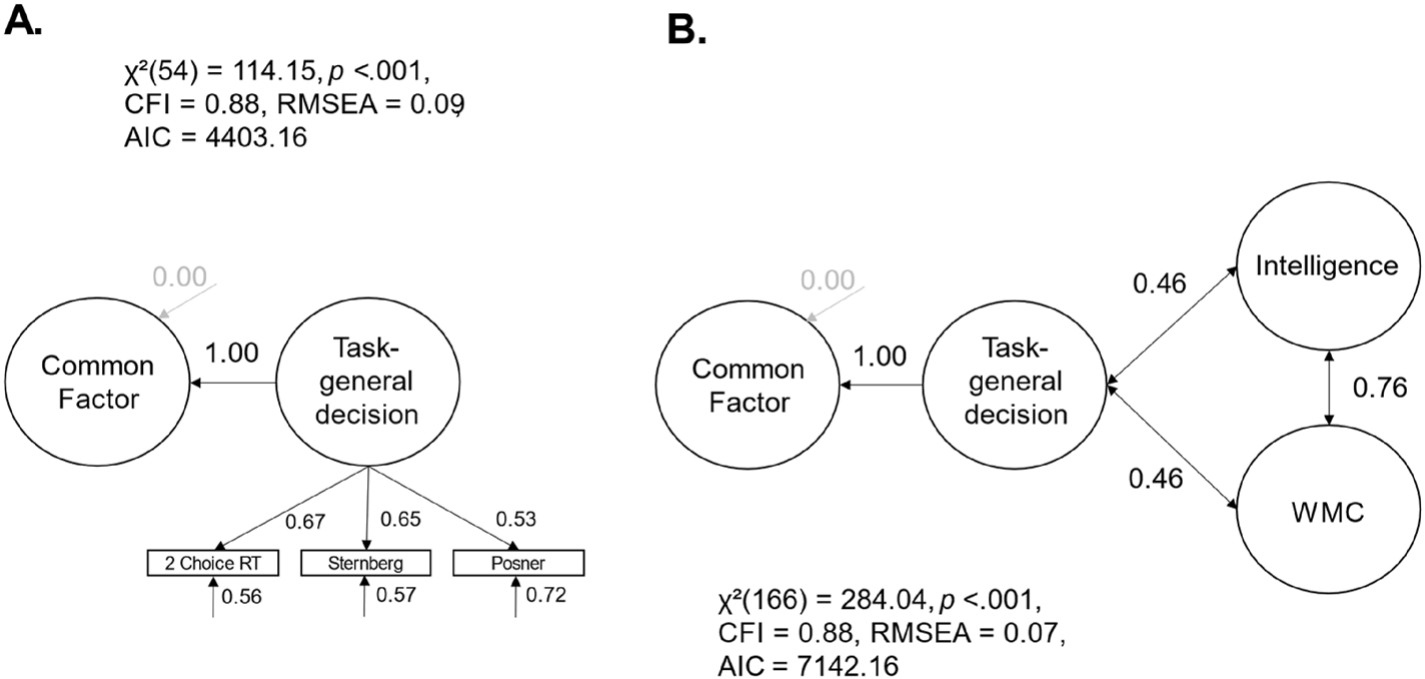
Models with one common factor of executive controlled for task-general speed of information uptake and its correlations to higher-order cognitive abilities (drift rates as homogenous measurement scores). *Note. *The standardized path and regression weights, the unstandardized residual variances, and the correlation coefficients are shown next to the paths; non-significant estimators are grayed out.; WMC = working memory capacity

So far, the common variance of the drift rates represented both task-general speed of information uptake as well as variance specific to executive functions. In the next step, we wanted to assess if the relationship between the variance specific to executive functions and intelligence as well as WMC pertained if we controlled for task-general speed of information uptake by introducing a latent *speed* factor to the model. This speed factor represented the covariance of three elementary cognitive tasks. The common executive functions factor was regressed on the latent speed factor to account for individual differences in task-general speed of information uptake. The model provided an almost acceptable fit of the data, *χ*^2^(53) = 114.14, *p* < 0.001, CFI = 0.87, RMSEA = 0.09, 95% CI [0.06, 0.11]. However, there was no significant variance in the common executive functions factor independent of task-general speed of information uptake, σ^2^ = − 0.00, *p* = 0.913, 95% CI [− 0.07, 0.06]. Given the small negative and non-significant residual variance of the common executive functions factor, we followed the recommendations by Chen et al. (2012) and fixed this residual variance to zero. This hardly changed the model fit, ∆ AIC = 1.99, *χ*^2^(54) = 114.15, *p* < 0.001, CFI = 0.88, RMSEA = 0.09, 95% CI [0.06, 0.11] (see Fig. 5A). In sum, the common factor of executive functions was completely explained by the task-general speed of information uptake, β = 1.00, *p* < 0.001. In this context, the shared variance of executive function tasks as measured with drift rates only represented individual differences in task-general speed of information uptake.

Again, we included WMC and intelligence as latent factors into the model to examine the relations between task-general speed of information uptake and higher-order cognitive abilities (see Fig. 5B). The model provided an almost acceptable fit of the data, *χ*^2^(166) = 284.04, *p* < 0.001, CFI = 0.88, RMSEA = 0.07, 95% CI [0.05, 0.09]. Participants’ task-general speed of information uptake was moderately correlated with intelligence (*r* = 0.46, *p* < 0.001, 95% CI [0.29, 0.63]) and WMC (*r* = 0.46, *p* < 0.001, 95% CI [0.31, 0.62]).^4^ Taken together, we found that executive functions measured by drift rates revealed one latent factor of common executive functions. This factor was completely explained by individual differences in task-general speed of information uptake. No variance specific to executive functions remained in our model, which could not be explained by the general speed of information uptake. Participants with a higher general speed of information uptake showed better performance in each of the nine executive function tasks and revealed higher intelligence test scores as well as greater WMC.

## Discussion

The aim of the present study was to examine the factor structure of the three executive functions as described by Miyake et al. (2000) using the drift rate parameter of the drift–diffusion model as a homogenous measurement score instead of heterogeneous measurement scores.

### Heterogenous measurement scores: a partial replication of the three-factor model

As a first step, we tried to replicate the original model of three distinct but interrelated factors with heterogeneous measurement scores, which was nearly identical to the model proposed in the seminal paper by Miyake et al. (2000). However, using RT difference scores, it was not possible to find significant variance for the factor of inhibition, that is, we did not find a coherent structure of the inhibition construct. This is in line with some recent research, suggesting that inhibition tasks often do not reveal a homogeneous structure of the underlying construct if one uses difference scores as performance measures from different tasks (Frischkorn & von Bastian, 2021; Hull et al., 2008; Krumm et al., 2009; Rey-Mermet et al., 2018; Stahl et al., 2014). When we used the mean RTs of the incongruent conditions of inhibition tasks, as done in several previous studies (see von Bastian et al., 2020), we found a coherent latent inhibition factor and were able to replicate the three-factor structure of executive functions by Miyake et al. (2000) with substantial correlations between these factors. However, such condition-specific scores suffer from validity problems because they may also be affected by task-general processes.

### The model of executive functions measured with drift rates

We used drift rates of the drift–diffusion model as homogenous measurement scores and structural equation models to separate task-general properties of drift rates from properties specific to executive functions. Our approach avoided the use of difference scores and their psychometric problems, thereby overcoming the two main issues of measuring executive functions—inconsistent use of RT- and accuracy-based scores and psychometric problems of difference scores. We found three latent factors of executive functions that loaded on one higher-level common executive functions factor. After introducing the higher-order factor, we observed no remaining variance specific to the three executive functions on the first-order latent level. We therefore specified a more parsimonious one-factorial solution, in which all tasks loaded on one latent first-order common executive functions factor. This model described the data only marginally worse than the more complex hierarchical model (∆ AIC = 7.60) and provided a good account of the data. In addition, given the absence of significant residual variances in the hierarchical model, the additional explanatory power of the more complex model seems highly questionable. In this context, the one-factor solution emerges as the more favorable model. However, future work should replicate our results with a larger sample size to get a better understanding of the nature of the common factor and the very small non-significant executive function specific variances as shown in Fig. 3B.

These findings, of a one-factor model, are in contrast to the three-factor model proposed by Miyake et al. (2000) and several other papers that found distinct factors of executive functions by using heterogeneous scoring methods (e.g., Friedman et al., 2006, 2008; Himi et al., 2019, 2021; Ito et al., 2015; Krumm et al., 2009; Schnitzspahn et al., 2013; Vaughan & Giovanello, 2010; Wongupparaj et al., 2015). Nevertheless, our finding of one common factor is consistent with previous results by Weigard et al. (2021), who also used drift rates and found that different cognitive control tasks loaded on only one common factor.

In the next step, we controlled for task-general processes included in drift rates (i.e., general speed of information uptake) and found that the latent common executive functions factor was fully accounted for by the task-general speed information uptake factor. In this model, no variance specific to executive functions remained. Hedge et al. (2022) found that inhibition tasks account for little common variance in inhibition processes but reflect consistent differences in task-general processing speed. They concluded that executive function processes should only be interpreted after controlling for task-general processes.Our results confirm the call by Hedge et al. (2022) to control the common variance among executive function tasks for task-general processes, suggesting that the observed common variance in the nine executive function tasks reflects nothing more than differences in the basic speed of information uptake. This is consistent with previous findings indicating that tasks supposedly measuring executive functions largely capture individual differences in the speed of information processing (Frischkorn & von Bastian, 2021; Frischkorn et al., 2019).

At this point we want to emphasize that drift rates are appropriate measures to separate task-general processes from domain-specific processes, and that these do not generally only yield one common speed of information uptake factor. In a recent paper, Lerche et al. (2020) demonstrated that drift rates can reflect different domain-general and domain-specific processes by showing that drift rates estimated from a battery of RT tasks differing in their complexity and in their content (figural, numeric, and verbal) reflected distinct factors of task-general as well as complexity- and content-specific variances. These results show that drift rates do not only measure the basic speed of information uptake, but that they may also reflect distinct processes. In consequence, our finding that drift rates in executive function tasks only represent task-general speed of information uptake is not a methodological artifact. Instead, it indicates that executive function tasks measure almost exclusively differences in basic speed of information uptake. Thus, executive function factors observed in previous studies likely only reflected individual differences in general processing speed.

Furthermore, we found that individual differences in general speed of information uptake were moderately correlated to intelligence and WMC. It is well known that information processing speed in elementary cognitive tasks is related to cognitive abilities (Doebler & Scheffler, 2016; Schubert et al., 2015, 2017, 2022b; Sheppard & Vernon, 2008). Several papers showing correlations between higher-order cognitive abilities and executive functions using heterogenous measurement scores (Benedek et al., 2014; Conway et al., 2021; Friedman et al., 2006, 2008; Wongupparaj et al., 2015) may have overestimated the relation between executive functions and higher-order cognitive abilities, because it is plausible that they largely estimated the relations between information processing speed and higher-order cognitive abilities.

### Implications for future research on executive functions

From our findings we derive three possible consequences: First, there may be no individual differences in cognitive abilities that are specific for executive functions. Second, it may be necessary to think about the coherence of specific executive functions on a theoretical level. Third, many of the indicator scores and tasks used so far may be inappropriate to capture individual differences in executive functions.

Our first conclusion is contrary to experiences we make in daily life. Every day, we experience situations in which we have the feeling that we are using executive processes. We must ignore irrelevant or distracting information to navigate traffic safely, we must update our memory content when playing a memory card matching game, and, when multitasking, we must shift between different tasks. As already mentioned at the beginning of the introduction, we consider executive functions as abilities. If executive functions exist in the sense of abilities, we have to assume that people differ in these abilities. The word “ability” is defined “as the quality or state of being able” (Merriam-Webster, 2022, 13. July), which is characterized by variation, because qualities and states vary between individuals. It is also well known that an extremely low level of executive abilities is associated with unfavorable or pathological outcomes, as—for example—extremely low inhibition abilities are associated with attention-deficit/hyperactivity disorder (ADHD; e.g., Wodka et al., 2007). In consequence, the dual-pathway model of ADHD describes poor inhibitory control as a central aspect of ADHD symptoms (Sonuga-Barke, 2002). Taken together, it is hard to believe that people do not differ in their executive functions.

Perhaps we should reconsider the coherence of specific executive functions on the theoretical level. Von Bastian et al. (2020) showed in their review that executive function tasks yield on average only small correlations among each other (median *r* = 0.16). Specifically, inhibition tasks did usually show absent or very small correlations with each other (see von Bastian et al., 2020), which suggests that inhibition may be needed to be defined more precisely and possibly be split into distinct abilities. Rey-Mermet et al. (2018) already demonstrated that two distinct abilities of inhibition exist, the inhibition of prepotent responses and the inhibition of distractor interference. However, a subsequent study could not replicate the proposed two-factorial solution with adequate model fit (Gärtner & Strobel, 2021). In our study, the inhibition tasks showed only small to absent correlations (from *r* = 08. to *r* = 0.17) when measured with RT-differences scores. This suggests that the executive processes contributing to performance in the inhibition tasks in our study may reflect distinct abilities, which could be one reason for why we did not find a coherent factor of inhibition when using RT-difference scores as indicator variables. Moreover, it is possible that the other executive functions also reflect a more differentiated pattern of the underlying abilities. Future research should reflect the divergence of executive functions on a theoretical level.

Alternatively, executive function tasks may be inappropriate to capture individual differences in executive functions. In our study, task-general speed of information uptake fully accounted for the shared variance between different executive function tasks. It seems that the classical executive function tasks capture to large parts task-general processes and no variance specific to executive functions. Therefore, we as a field should create new tasks or develop new cognitive mathematical models to better measure individual differences in these executive function abilities.

#### The development of new tasks to measure executive function abilities more validly

Recent studies have proposed developing and modifying executive function tasks to better capture individual differences in executive functions. Draheim et al. (2021) developed a battery of new and modified (Flanker and Stroop) inhibition tasks and compared them with different classical inhibition tasks. In the newly developed tasks, properties of the task (e.g., presentation time of the stimulus or the maximally allotted response time) adjusted dynamically as a function of participants’ performance in previous trials. If the performance was good enough, the presentation times of stimuli or response deadlines were lowered, otherwise they were raised. In this adaptive staircase procedure, the authors used in some tasks the individually calibrated presentation times and in other tasks the individually calibrated response deadlines as dependent variable. Draheim et al. (2021) found substantial intercorrelations between most of these tasks and subsequently a coherent latent factor of attentional control, which is a construct closely related to executive functions. In addition, this latent factor correlated with WMC and intelligence and these correlations could not be explained by task-general processing speed. Further research reported additional evidence for the validity of this battery of novel executive function tasks by finding a common factor of executive processes independent of task-general processing speed (Burgoyne et al., 2022; Draheim et al., 2023). These modified tasks were highly reliable (all estimates ≥ 0.86) and fast to administer (see: Burgoyne et al., 2022).

However, there is a particular aspect that must be taken into account when discussing our findings with regard to the framework proposed by Draheim et al., (2021, 2023). It is possible that the discrepancy between both studies regarding the correlations between executive function abilities and processing speed stems from differences in the conceptualization of the mental speed factors and the specific tasks used to measure mental speed. Draheim and colleagues typically employed tasks in which participants compared patterns of stimuli and decided whether two patterns were equal or different. In contrast, our tasks required participants to make elementary decisions based on a currently presented stimulus. Both sets of tasks can be considered as measures of mental speed. However, the focus lies on different aspects of mental speed. The distinction between both concepts can be illustrated based on the Cattell-Horn-Carroll (CHC) model (Carroll, 1993). In the CHC model, Draheim and colleagues’ tasks align with the processing speed factor (*Gs*), while our tasks align with the reaction and decision speed factor (*Gt*). Both of these factors belong to the CHC model’s broader abilities in Stratum II and are considered to have separate contributions to general intelligence (e.g., Carroll, 1993). Because our battery of elementary cognitive tasks corresponds to the abilities represented in Carrol’s *Gt* factor, the common factor of executive function tasks was completely explained by processing speed. All the variance shared among executive function tasks is essentially attributed to task-general information processing and decision-making abilities. In contrast, Draheim and colleagues’ elementary cognitive tasks are measuring perceptual and clerical speed. These differences in the conceptualizations between Draheim et al. (2021, 2023) and our lab may account for the different empirical observations. It remains unclear to which extent the cognitive control factor by Draheim et al. (2021, 2023) diverges from a speed factor when measured with our processing speed tasks.

Our findings shed alarming light on classical executive function tasks, revealing that the shared variance among these tasks primarily represents task-general processing and decision-making abilities. Nevertheless, the work by Draheim et al. (2021, 2023) demonstrates that the development of novel measures and new tasks are promising approaches to make progress in the research of measuring individual differences in executive functions. Because it would be important to demonstrate that their tasks do not only measure processing speed as measured by the tasks included in the present study, it is obvious that more research is needed.

#### Cognitive mathematical modeling approaches to measure executive function abilities more validly

In addition to novel tasks and measurement scores, cognitive mathematical modeling approaches could also be a promising approach to validly measure individual differences in executive functions. A recent study validated the model parameters of the dual-stage two-phase model by Hübner et al. (2010)—a specific cognitive model to capture inhibition abilities in the Arrow Flanker Task—with inhibition-related electrophysiological correlates and found meaningful correlations between model parameters and event-related potential components (Schubert et al., 2022a). Jointly, the process parameters explained 37% variance in higher-order cognitive abilities (Schubert et al., 2022a). However, the authors did not control the model parameters for the influence of task-general processes. It therefore remains open to which degree the parameters reflected task-general processing efficiency. Nevertheless, the findings by Schubert et al., (2022a) demonstrate that cognitive mathematical models could be a fruitful way to capture individual differences in executive abilities. The use of cognitive mathematical model parameters and the development of specific cognitive mathematical models should be further promoted in the field of executive functions research. However, it is necessary to demonstrate that model parameters possess divergent validity to basic speed of information uptake.

### Limitations

One major limitation of the diffusion modeling approach implemented in the present study is that the standard drift–diffusion model is not ideally suited to model RT distributions associated with incorrect responses in inhibition tasks. Because the drift rate is assumed to be constant over the course of a single trial, it is unable to account for the characteristic data pattern observed in conflict tasks, specifically the occurrence of faster errors in incongruent trials compared to correct responses (White et al., 2011). To address this limitation of the standard drift–diffusion model, models with time-varying drift rates like the diffusion model for conflict tasks (Ulrich et al., 2015), the dual-stage two-phase model (Hübner et al., 2010), and the shrinking spotlight model (White et al., 2011) have been developed. Since these models assume drift rates that change over time, they are more appropriate to account for the characteristic data pattern observed in conflict tasks. However, in the present study, it was not feasible to estimate these models with time-varying drift rates instead of the standard diffusion model, as we included not only conflict (inhibition) tasks but also updating and shifting tasks in our study. As a result, these models with time-varying drift rates could only be applied to a subset of our data and not to data from all nine executive function tasks. Nonetheless, we have confidence that our conclusions were not influenced by using the standard diffusion model, as our findings align with those of a previous study that used the diffusion model for conflict tasks to estimate the correlation of conflict-related model parameters across four different conflict tasks (Hedge et al., 2022). Consistent with our results, this study also found only very low and statistically insignificant correlations across tasks, although these correlations may have been underestimated due to the low reliabilities of control-related model parameters.

Another limitation pertaining to the estimation of the diffusion model in the present study is that *v* reflects both RTs and accuracies jointly, since the RT distributions of both correct and incorrect responses are used for its estimation. However, this was not the case for all tasks in our study. For the Two Choice Reaction Time task, the drift rate parameter mainly reflected RT variance because accuracy rates were near ceiling (i.e., there was virtually no distribution of incorrect responses, mean correct responses 99.47%, *SD* = 0.98%). That is why the parameter recovery revealed no correlation between predicted and observed accuracies (*r* = 0.05). In comparison, the quantiles of the RT-distribution of the Two Choice Reaction Time task were recovered with high precision (range from *r* = 0.98 to *r* = 0.99; see also Fig. S4 in the supplementary materials). Nevertheless, the drift rates of the Two Choice Reaction Time task were substantially correlated with the drift rates of both other elementary cognitive tasks, *r* = 0.35 to *r* = 0.37. These manifest correlations were comparable to the correlation between the drift rates in those two other tasks (*r* = 0.39) and indicate that the drift rates of the Two Choice Reaction Time task showed convergent validity to the drift rates of the two other elementary cognitive tasks. For the other 11 tasks, we observed correlations between *r* = 0.47 to *r* = 0.99 for RTs and accuracies between predicted and observed scores. All in all, we can be relatively certain that our models yielded valid parameter estimates.

Finally, we examined a sample of *N* = 148 participants, which is a sufficient sample size as we needed a minimum sample size of *N* = 95 to test the hypothesis of close fit. However, given the uncertainty of correlations, examining larger groups of individuals would strengthen the robustness of our correlational findings (Kretzschmar & Gignac, 2019; Schönbrodt & Perugini, 2013). Therefore, future research should try to replicate the absolute magnitude of correlations in our study as their estimations had a relatively large degree of uncertainty.

## Conclusion

In our present study, we examined the factor structure of the three executive functions by Miyake et al. (2000). We used a cognitive mathematical modeling approach to overcome the problems associated with the inconsistent use of accuracy vs. RT-based scores as indicator variables and the use of manifest difference scores, which can sometimes cause psychometric problems. Applying the drift–diffusion model, we found a one-factorial structure of executive function tasks. However, in this analysis, we used only the drift rates from the conditions with greater processing demands. Because drift rates in these conditions were affected by both task-general and executive function processes, the latent common executive functions factor reflected individual differences in both types of processes. After controlling for individual differences in these task-general processes, we observed no unique variance specific to executive functions. This indicates that the covariance between different executive function tasks can be fully accounted for by individual differences in the general speed of information uptake, which was moderately related to higher-order cognitive abilities. Applying this drift–diffusion model account thus shed alarming light on tasks supposedly measuring executive functions. We observed no variance specific to executive functions that was independent of the general speed of information uptake. Thus, the development or modification of executive function tasks is necessary to capture individual differences in executive functions reliably and validly, assuming that such differences exist.

### Supplementary Information

Below is the link to the electronic supplementary material.

Supplementary file1 (DOCX 193 KB)

## Data Availability

We provide access to the preprocessed data and the statistical analysis code used for this paper via the Open Science Framework (https://osf.io/6c4pu/). In addition, we provide access to the raw data and to the materials via the Open Science Framework (https://osf.io/4pvz3/; except for the materials of the BIS, which are commercially licensed). Neither the study nor the analyses were preregistered.

## Footnotes

1 We consider cognitive control as a construct that is closely related to executive functions.

2 We followed the recommendations by MacCallum et al. (1996) and conducted the power analysis by comparing the null hypothesis RMSEA (RMSEA = .05) with an alternative hypothesis RMSEA (RMSEA = .08). Using both RMSEA values, the given sample size of *N* = 148, and the degrees of freedom of the model (e.g., *df* = 166), we calculated the non-centrality parameters for both hypotheses. With these parameters, an α = .05, and the given *df*s, we calculated the critical χ^2^ value and subsequently the observed power using the cumulative distribution function of the χ^2^ distribution.

3 If we introduced a higher-order cognitive abilities factor in our model instead of intelligence and WMC separately, the general executive functions factor was also moderately related to cognitive abilities, *r* = .48, *p* < .001, 95% CI [.31, .66].

4 If we introduced a higher-order cognitive abilities factor in our model instead of intelligence and WMC separately, the correlation between higher-order cognitive abilities and task-general speed of information uptake was also high, *r* = .53, *p* < .001, 95% CI [.37, .69].
