# Supplementary material for: The common factor of executive functions measures nothing but speed of information uptake
Source: Psychol Res. 2024 Feb 19;88(4):1092–114. doi: 10.1007/s00426-023-01924-7 (PMC11143038; doi:10.1007/s00426-023-01924-7)
Supplement: Supplementary file 1 — Supplementary file1 (DOCX 193 KB) [file 426_2023_1924_MOESM1_ESM.docx]

**Supplementary materials**

**Table S1** *Presentation times of the memory-set stimuli, probe stimuli, and intervals between stimuli and trials in the different tasks*

| Task name | Process | Fixation cross | Inter-stimulus interval | Memory presentation time | Cue presentation time | Probe presentation time | Inter-trial interval |
| --- | --- | --- | --- | --- | --- | --- | --- |
| Negative priming task | inhibition | 400 - 600 ms | 400 - 600 ms | --- | --- | 1000 - 3000 ms | 1000 - 1500 ms |
| Flanker task | inhibition | 400 - 600 ms | 400 - 600 ms | --- | --- | 1000 - 3000 ms | 1000 - 1500 ms |
| Stroop task | inhibition | 400 - 600 ms | 400 - 600 ms | --- | --- | 1000 - 3000 ms | 1000 - 1500 ms |
| Keep track task | updating | 400 - 600 ms | 400 - 600 ms | 1000 ms | 800 - 1200 ms | 1000 - 3000 ms | 1000 - 1500 ms |
| Running span task | updating | 400 - 600 ms | 400 - 600 ms | 1000 ms | 800 - 1200 ms | 1000 - 3000 ms | 1000 - 1500 ms |
| N-Back task | updating | --- | 400 - 600 ms | --- | --- | 1500 ms | --- |
| Number-letter task | shifting | 400 - 600 ms | 400 - 600 ms | --- | --- | 1000 - 3000 ms | 1000 - 1500 ms |
| Switching task | shifting | 400 - 600 ms | 400 - 600 ms | --- | --- | 1000 - 3000 ms | 1000 - 1500 ms |
| Global-local task | shifting | 400 - 600 ms | 400 - 600 ms | --- | --- | 1000 - 3000 ms | 1000 - 1500 ms |
| Two-choice-RT task | speed | 1000 - 1500 ms | --- | --- | --- | 1000 - 3000 ms | 1000 - 1500 ms |
| Sternberg task | speed | 1000 - 1500 ms | 400 - 1000 ms | 1000 ms | 1800 - 2200 ms | 1000 - 3000 ms | 1000 - 1500 ms |
| Posner task | speed | 1000 - 1500 ms | --- | --- | --- | 1000 - 3000 ms | 1000 - 1500 ms |
| *Note.* Probe stimuli were presented until participants responded; If participants' response was faster than 1000 ms the stimulus remained till 1000 ms were reached; The stimulus disappeared after 3000 ms if the participants did not respond (after 1500 ms in the N-Back task); ms = milliseconds. | | | | | | | |

**Table S2** *Correlations between all variables (heterogeneous* *measurement scores)*

|  |  | 1 | 2 | 3 | 4 | 5 | 6 | 7 | 8 | 9 | 10 | 11 | 12 | 13 | 14 | 15 | 16 | 17 | 18 | 19 | 20 | 21 | 22 |
| --- | --- | --- | --- | --- | --- | --- | --- | --- | --- | --- | --- | --- | --- | --- | --- | --- | --- | --- | --- | --- | --- | --- | --- |
| 1 | Negative Pr. task, RT difference |  |  |  |  |  |  |  |  |  |  |  |  |  |  |  |  |  |  |  |  |  |  |
| 2 | Flanker task, RT difference | **.17** |  |  |  |  |  |  |  |  |  |  |  |  |  |  |  |  |  |  |  |  |  |
| 3 | Stroop task, RT difference | .08 | **.15** |  |  |  |  |  |  |  |  |  |  |  |  |  |  |  |  |  |  |  |  |
| 4 | Negative Pr. task, RT priming cond. | **.43** | **.20** | **.17** |  |  |  |  |  |  |  |  |  |  |  |  |  |  |  |  |  |  |  |
| 5 | Flanker task, RT incong. cond. | **.22** | **.42** | **.23** | **.72** |  |  |  |  |  |  |  |  |  |  |  |  |  |  |  |  |  |  |
| 6 | Stroop task, RT incong. cond. | **.25** | **.20** | **.60** | **.67** | **.59** |  |  |  |  |  |  |  |  |  |  |  |  |  |  |  |  |  |
| 7 | Keep Track, prop. updating cond. | -.05 | -.08 | .09 | **-.34** | **-.20** | **-.21** |  |  |  |  |  |  |  |  |  |  |  |  |  |  |  |  |
| 8 | Running Span, prop. updating cond. | -.01 | -.08 | .04 | **-.20** | -.10 | -.11 | **.43** |  |  |  |  |  |  |  |  |  |  |  |  |  |  |  |
| 9 | N-Back task prop | -.09 | **-.17** | .02 | **-.30** | **-.24** | **-.22** | **.35** | **.32** |  |  |  |  |  |  |  |  |  |  |  |  |  |  |
| 10 | Number Letter task, RT difference | .01 | .01 | .09 | .15 | **.25** | **.23** | -.11 | -.09 | **-.17** |  |  |  |  |  |  |  |  |  |  |  |  |  |
| 11 | Switching task, RT difference | .13 | .12 | **.18** | **.22** | **.19** | **.24** | **-.21** | .01 | **-.28** | **.45** |  |  |  |  |  |  |  |  |  |  |  |  |
| 12 | Global Local task, RT difference | .00 | .09 | .15 | **.17** | **.23** | **.25** | -.12 | -.07 | -.13 | **.44** | **.41** |  |  |  |  |  |  |  |  |  |  |  |
| 13 | Two Choice RT task, RT | **.17** | .11 | .16 | **.71** | **.66** | **.52** | **-.25** | -.08 | **-.21** | .09 | .12 | **.22** |  |  |  |  |  |  |  |  |  |  |
| 14 | Sternberg task, RT | .14 | **.19** | .10 | **.60** | **.64** | **.41** | **-.26** | -.14 | **-.33** | .14 | **.21** | **.22** | **.53** |  |  |  |  |  |  |  |  |  |
| 15 | Posner task, RT | **.18** | .06 | .15 | **.74** | **.62** | **.54** | **-.42** | **-.27** | **-.29** | **.17** | **.22** | .12 | **.70** | **.59** |  |  |  |  |  |  |  |  |
| 16 | Memory updating | -.08 | -.16 | .03 | **-.30** | **-.38** | **-.20** | **.25** | **.46** | **.42** | -.15 | -.05 | -.17 | **-.24** | **-.33** | **-.38** |  |  |  |  |  |  |  |
| 17 | Binding | **-.18** | -.16 | .10 | **-.34** | **-.38** | **-.22** | **.25** | **.32** | **.35** | -.07 | -.02 | -.10 | **-.22** | **-.40** | **-.31** | **.70** |  |  |  |  |  |  |
| 18 | Operation span | .03 | .01 | .09 | -.12 | **-.24** | -.06 | .09 | **.28** | **.26** | -.03 | .00 | -.07 | **-.19** | **-.29** | **-.18** | **.58** | **.50** |  |  |  |  |  |
| 19 | Sentence span | .03 | .03 | .04 | -.06 | -.12 | -.05 | .14 | **.33** | **.24** | -.12 | .02 | -.09 | -.14 | -.14 | -.16 | **.61** | **.35** | **.66** |  |  |  |  |
| 20 | BIS-PC | -.04 | -.02 | -.08 | **-.26** | **-.25** | **-.23** | **.23** | **.31** | **.43** | -.05 | -.13 | -.05 | -.16 | **-.31** | **-.36** | **.60** | **.53** | **.38** | **.41** |  |  |  |
| 21 | BIS-PS | -.08 | -.09 | -.02 | **-.36** | **-.39** | **-.28** | **.33** | **.25** | **.41** | -.16 | -.13 | **-.20** | **-.34** | **-.35** | **-.38** | **.42** | **.44** | **.20** | **.22** | **.50** |  |  |
| 22 | BIS-M | .08 | -.13 | -.02 | -.13 | **-.21** | **-.25** | **.20** | **.26** | **.34** | -.15 | **-.22** | **-.24** | -.10 | -.12 | **-.20** | **.34** | **.34** | **.23** | **.27** | **.43** | **.43** |  |
| 23 | BIS-C | -.01 | -.07 | -.13 | **-.21** | -.11 | -.08 | .05 | .08 | .12 | .04 | -.02 | .08 | -.03 | **-.17** | **-.20** | .13 | **.19** | .09 | .13 | **.35** | **.34** | .13 |

*Note.* Heterogeneous measurement scores; BIS-PC = processing capacity scale of the Berlin Intelligence Structure Test; BIS-PS = processing speed scale of the Berlin Intelligence Structure Test; BIS-M = memory scale of the Berlin Intelligence Structure Test; BIS-C = creativity scale of the Berlin Intelligence Structure Test; 1-3 RT difference scores; 4-6 mean RT of the incongruent condition; 7-9 arcsine transformed proportion correct scores; 10-12 RT difference scores; 13-15 mean RT; 16-19 percentage correct; 20-23 scale scores; Significant correlations (*p* < .05) are presented in bold.

**Table S3** *Descriptive statistics of the drift-diffusion model parameters*

|  | *a* mean | *a* *SD* | *v* mean | *v* *SD* | *t_0_* mean | *t_0_* *SD* | *st_0_* mean | *st_0_* *SD* |
| --- | --- | --- | --- | --- | --- | --- | --- | --- |
| Negative priming task, priming cond. | 1.28 | 0.29 | 3.62 | 0.94 | 0.42 | 0.07 | 0.13 | 0.08 |
| Flanker task, incong. cond. | 1.05 | 0.29 | 5.05 | 1.32 | 0.40 | 0.05 | 0.12 | 0.05 |
| Stroop task, incong. cond. | 1.44 | 0.37 | 2.60 | 0.74 | 0.55 | 0.12 | 0.28 | 0.15 |
| Keep track, updating cond. | 1.72 | 0.34 | 1.69 | 0.62 | 0.41 | 0.17 | 0.27 | 0.24 |
| Running span, updating cond. | 1.49 | 0.40 | 1.78 | 0.58 | 0.53 | 0.12 | 0.18 | 0.18 |
| N-Back task | 1.47 | 0.22 | 1.74 | 0.46 | 0.36 | 0.11 | 0.25 | 0.17 |
| Number letter task, shifting cond. | 1.67 | 0.43 | 2.29 | 0.92 | 0.39 | 0.15 | 0.18 | 0.26 |
| Switching task, shifting cond. | 1.74 | 0.44 | 2.16 | 0.85 | 0.37 | 0.11 | 0.23 | 0.26 |
| Global local task, shifting cond. | 2.01 | 0.37 | 1.65 | 0.51 | 0.62 | 0.25 | 0.44 | 0.40 |
| Two choice RT task | 0.95 | 0.24 | 6.25 | 1.58 | 0.30 | 0.04 | 0.08 | 0.05 |
| Sternberg task | 1.52 | 0.41 | 2.35 | 0.71 | 0.61 | 0.16 | 0.24 | 0.16 |
| Posner task | 1.38 | 0.35 | 3.27 | 0.74 | 0.49 | 0.07 | 0.19 | 0.09 |

*Note.* All estimated drift-diffusion model parameters are displayed in this table; *a =* boundary separation parameter; *v =* drift parameter; *t_0_ =* non-decision time parameter; *st_0_* = inter-trial variability of non-decisional components.

**Table S4** *Correlations between all variables (drift rates v as homogenous measurement scores)*

|  |  | 1 | 2 | 3 | 4 | 5 | 6 | 7 | 8 | 9 | 10 | 11 | 12 | 13 | 14 | 15 | 16 | 17 | 18 | 19 |
| --- | --- | --- | --- | --- | --- | --- | --- | --- | --- | --- | --- | --- | --- | --- | --- | --- | --- | --- | --- | --- |
| 1 | Negative Pr. task, priming cond. |  |  |  |  |  |  |  |  |  |  |  |  |  |  |  |  |  |  |  |
| 2 | Flanker task, incong. cond. | **.32** |  |  |  |  |  |  |  |  |  |  |  |  |  |  |  |  |  |  |
| 3 | Stroop task, incong. cond. | .05 | **.30** |  |  |  |  |  |  |  |  |  |  |  |  |  |  |  |  |  |
| 4 | Keep Track, updating cond. | **.34** | **.29** | .07 |  |  |  |  |  |  |  |  |  |  |  |  |  |  |  |  |
| 5 | Running span, updating cond. | **.36** | **.24** | .04 | **.35** |  |  |  |  |  |  |  |  |  |  |  |  |  |  |  |
| 6 | N-Back task | **.27** | .15 | -.01 | **.33** | **.26** |  |  |  |  |  |  |  |  |  |  |  |  |  |  |
| 7 | Number Letter task, shifting cond. | **.39** | **.34** | **.21** | **.38** | **.24** | **.24** |  |  |  |  |  |  |  |  |  |  |  |  |  |
| 8 | Switching task, shifting cond. | **.42** | **.35** | **.34** | **.42** | **.35** | **.26** | **.60** |  |  |  |  |  |  |  |  |  |  |  |  |
| 9 | Global Local task, shifting cond. | **.18** | **.37** | **.33** | .17 | **.26** | **.26** | **.47** | **.53** |  |  |  |  |  |  |  |  |  |  |  |
| 10 | Two Choice RT task | **.34** | **.44** | **.21** | **.38** | **.36** | **.21** | **.45** | **.57** | **.29** |  |  |  |  |  |  |  |  |  |  |
| 11 | Sternberg task | **.30** | **.34** | .13 | **.49** | **.53** | **.41** | **.44** | **.38** | **.35** | **.37** |  |  |  |  |  |  |  |  |  |
| 12 | Posner task | **.34** | **.34** | .14 | **.39** | **.36** | **.25** | **.32** | **.31** | .15 | **.35** | **.39** |  |  |  |  |  |  |  |  |
| 13 | Memory updating | **.17** | .11 | .02 | **.25** | **.46** | **.38** | .16 | **.31** | **.25** | **.24** | **.43** | **.23** |  |  |  |  |  |  |  |
| 14 | Binding | **.19** | .03 | -.12 | **.32** | **.29** | **.27** | .03 | **.22** | .09 | **.19** | **.24** | **.23** | **.70** |  |  |  |  |  |  |
| 15 | Operation span | .02 | -.02 | .01 | .14 | **.32** | **.24** | .02 | **.24** | .02 | .10 | **.29** | .03 | **.58** | **.50** |  |  |  |  |  |
| 16 | Sentence span | -.02 | .03 | .04 | .05 | **.23** | .17 | .15 | **.25** | .15 | .09 | **.24** | .05 | **.61** | **.35** | **.66** |  |  |  |  |
| 17 | BIS-PC | .14 | **.17** | .14 | **.22** | **.23** | **.31** | .07 | **.23** | **.18** | .16 | **.27** | **.27** | **.60** | **.53** | **.38** | **.41** |  |  |  |
| 18 | BIS-PS | .16 | .15 | .05 | **.28** | .17 | **.44** | .12 | **.32** | **.20** | **.21** | **.25** | **.21** | **.42** | **.44** | **.20** | **.22** | **.50** |  |  |
| 19 | BIS-M | .14 | **.19** | .16 | .14 | .14 | **.20** | .11 | **.32** | **.22** | .12 | .16 | .14 | **.34** | **.34** | **.23** | **.27** | **.43** | **.43** |  |
| 20 | BIS-C | .12 | .01 | .05 | .03 | .00 | .11 | -.01 | .08 | -.01 | .07 | .11 | .05 | .13 | **.19** | .09 | .13 | **.35** | **.34** | .13 |

*Note.* Drift rates *v* as homogenous measurement scores; BIS-PC = processing capacity scale of the Berlin Intelligence Structure Test; BIS-PS = processing speed scale of the Berlin Intelligence Structure Test; BIS-M = memory scale of the Berlin Intelligence Structure Test; BIS-C = creativity scale of the Berlin Intelligence Structure Test; 1-12 drift rates *v*; 13-16 percentage correct; 17-20 scale scores; Significant correlations (*p* < .05) are presented in bold.

**Figure S1** *QQ-plots for the assessment of model fit based on the comparison of statistics (accuracy rate, 25%, 50%, and 75% quantile) of the observed and predicted data for inhibition tasks*


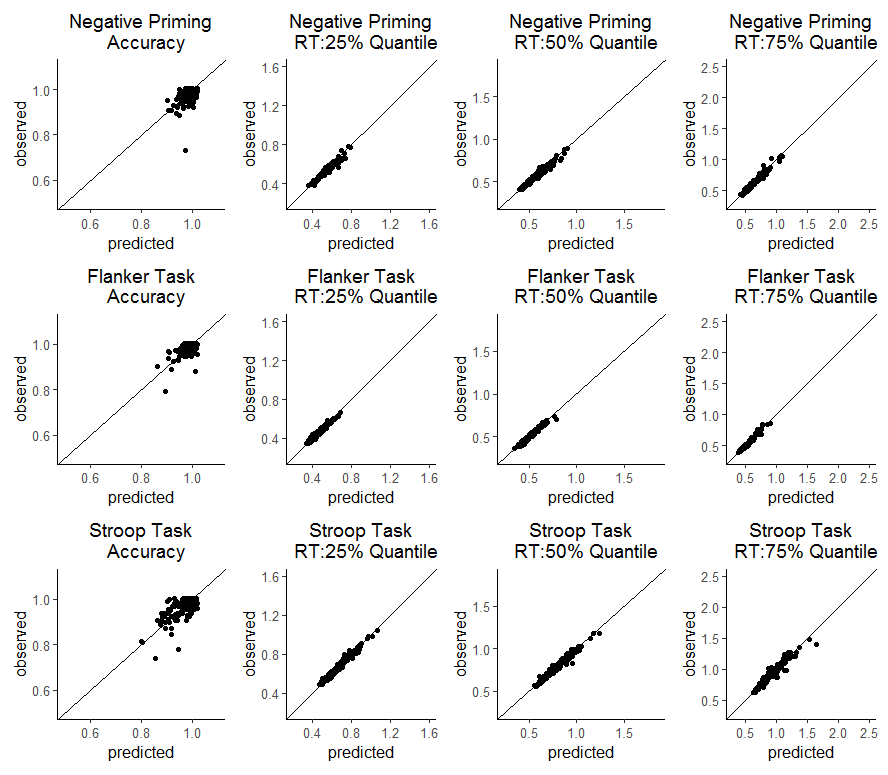


*Note.* Each data point represents one participant. The diagonal lines indicate perfect model fit.

**Figure S2** *QQ-plots for the assessment of model fit based on the comparison of statistics (accuracy rate, 25%, 50%, and 75% quantile) of the observed and predicted data for updating tasks*


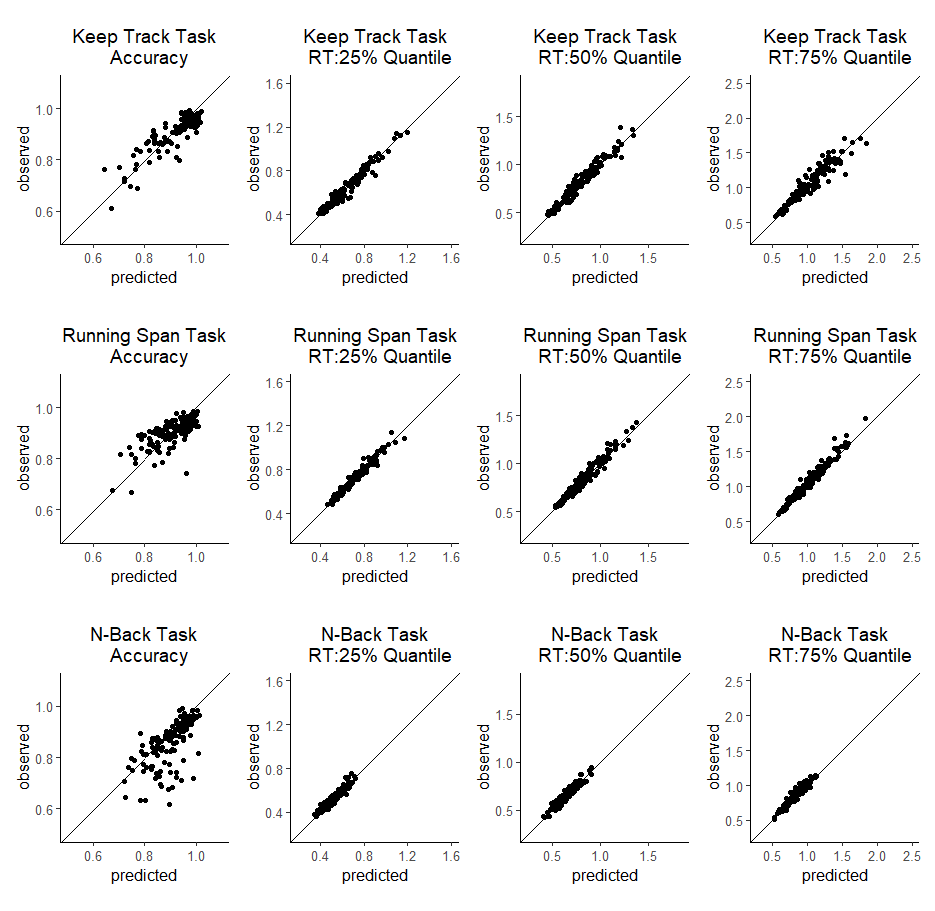


*Note.* Each data point represents one participant. The diagonal lines indicate perfect model fit.

**Figure S3** *QQ-plots for the assessment of model fit based on the comparison of statistics (accuracy rate, 25%, 50%, and 75% quantile) of the observed and predicted data for shifting tasks*


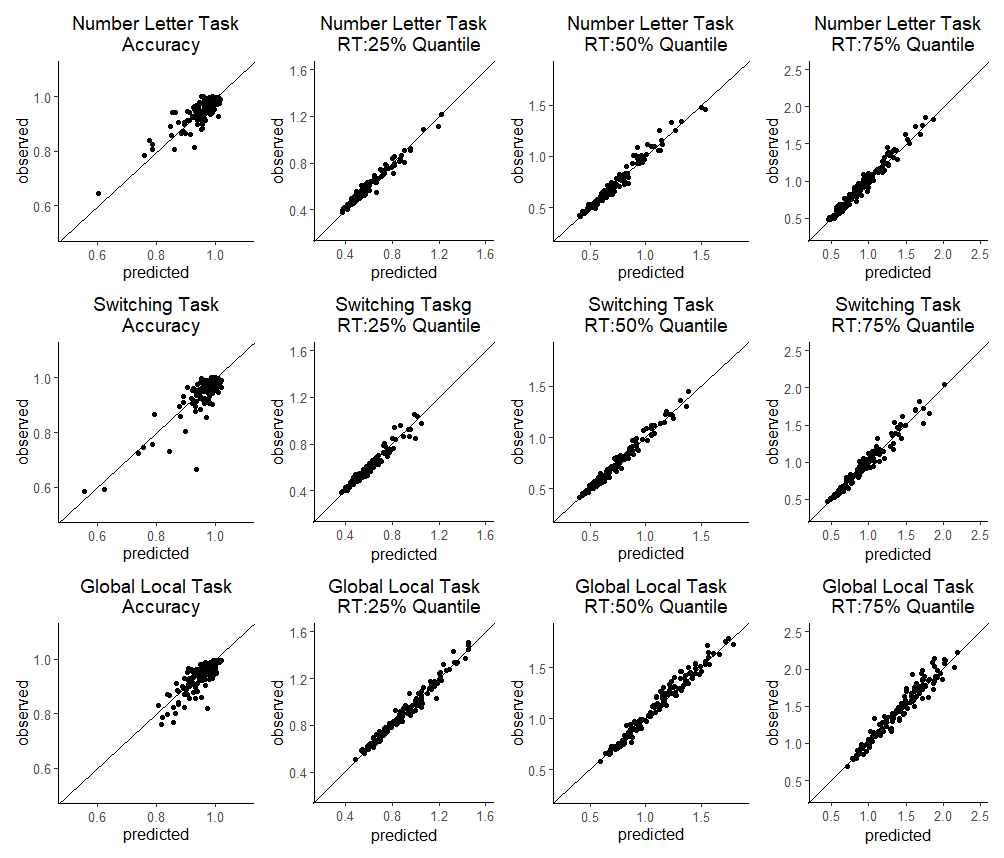


*Note.* Each data point represents one participant. The diagonal lines indicate perfect model fit.

**Figure S4** *QQ-plots for the assessment of model fit based on the comparison of statistics (accuracy rate, 25%, 50%, and 75% quantile) of the observed and predicted data for elementary cognitive tasks (information processing speed)*


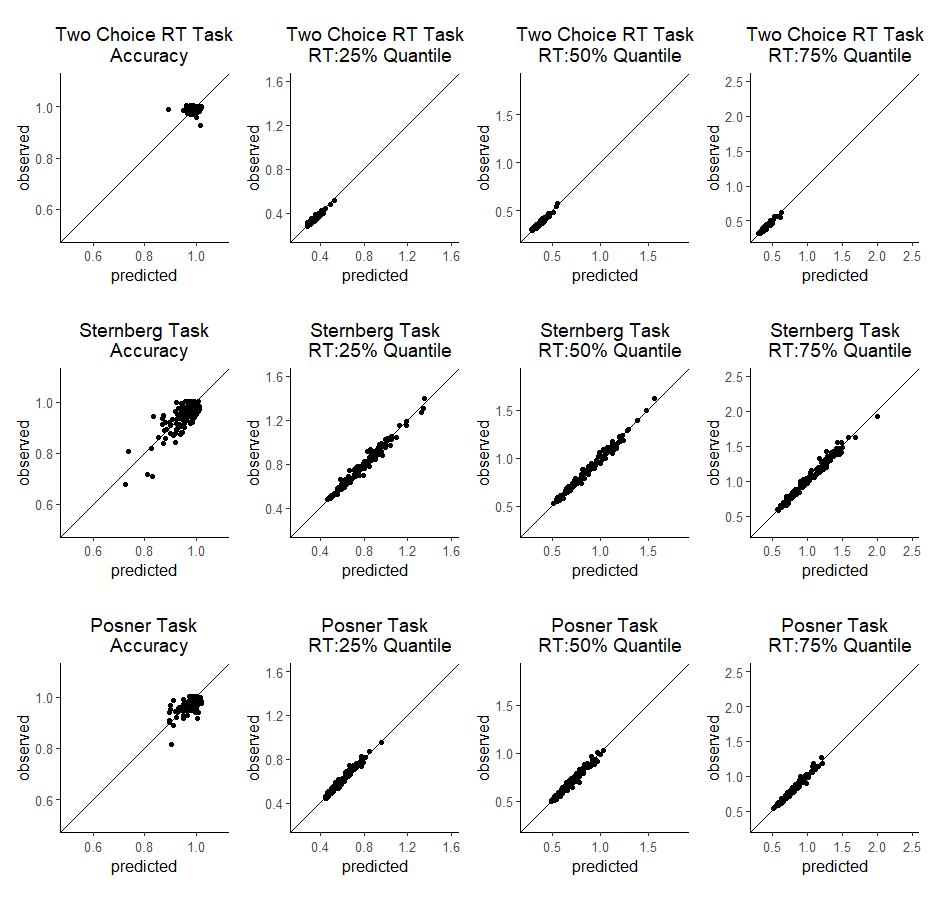


*Note.* Each data point represents one participant. The diagonal lines indicate perfect model fit.
